# It takes two to tango: cardiac fibroblast-derived NO-induced cGMP enters cardiac myocytes and increases cAMP by inhibiting PDE3

**DOI:** 10.1038/s42003-023-04880-5

**Published:** 2023-05-10

**Authors:** Lukas Menges, Jan Giesen, Kerem Yilmaz, Evanthia Mergia, Annette Füchtbauer, Ernst-Martin Füchtbauer, Doris Koesling, Michael Russwurm

**Affiliations:** 1 Institute of Pharmacology and Toxicology, Ruhr-University Bochum, 44780 Bochum, Germany; 2 Department of Molecular Biology and Genetics, Aarhus University, 8000 Aarhus C, Denmark

**Keywords:** Molecular medicine, Cardiovascular biology, Cell signalling

## Abstract

The occurrence of NO/cGMP signalling in cardiac cells is a matter of debate. Recent measurements with a FRET-based cGMP indicator in isolated cardiac cells revealed NO-induced cGMP signals in cardiac fibroblasts while cardiomyocytes were devoid of these signals. In a fibroblast/myocyte co-culture model though, cGMP formed in fibroblasts in response to NO entered cardiomyocytes via gap junctions. Here, we demonstrate gap junction-mediated cGMP transfer from cardiac fibroblasts to myocytes in intact tissue. In living cardiac slices of mice with cardiomyocyte-specific expression of a FRET-based cGMP indicator (αMHC/cGi-500), NO-dependent cGMP signals were shown to occur in myocytes, to depend on gap junctions and to be degraded mainly by PDE3. Stimulation of NO-sensitive guanylyl cyclase enhanced Forskolin- and Isoproterenol-induced cAMP and phospholamban phosphorylation. Genetic inactivation of NO-GC in Tcf21-expressing cardiac fibroblasts abrogated the synergistic action of NO-GC stimulation on Iso-induced phospholamban phosphorylation, identifying fibroblasts as cGMP source and substantiating the necessity of cGMP-transfer to myocytes. In sum, NO-stimulated cGMP formed in cardiac fibroblasts enters cardiomyocytes in native tissue where it exerts an inhibitory effect on cAMP degradation by PDE3, thereby increasing cAMP and downstream effects in cardiomyocytes. Hence, enhancing β-receptor-induced contractile responses appears as one of NO/cGMP’s functions in the non-failing heart.

## Abstract (web summary)

The use of living cardiac slices of mice demonstrates the occurrence of NO- and gap junction-dependent cGMP in myocytes increasing isoproterenol-induced cAMP and phospholamban phosphorylation, with fibroblasts as NO-dependent cGMP source.

## Introduction

Similar to the signalling molecule cAMP, cGMP is involved in the regulation of a multitude of physiological events^1^. Most of the cGMP-induced effects are mediated by activation of the cGMP-dependent protein kinases; in addition, cGMP-mediated inhibition of PDE3 (phosphodiesterase 3) or cGMP-induced PDE2 activation provides the molecular basis for a crosstalk with the cAMP signalling pathway. Likewise, cGMP-gated channels execute cGMP effects especially in the neuronal system. In general, two guanylyl cyclase families (GCs), the transmembrane guanylyl cyclases and the NO-sensitive guanylyl cyclase (NO-GC) catalyse the conversion of GTP to cGMP. Transmembrane guanylyl cyclases are activated by the natriuretic peptides ANP, BNP and CNP whereas NO-GC acts as the major receptor for the intra- and intercellular signalling molecule nitric oxide^2,3^.

The NO/cGMP pathway has an established function in the regulation of smooth muscle tone, inhibition of platelet aggregation and modulation of synaptic transmission^3^. Yet, in contrast to the relatively precise understanding of these NO/cGMP-induced events, the role of NO/cGMP in cardiac function is less clear and the functional consequences of cGMP increases have not been unequivocally established. For instance, high amounts of NO have been reported to induce negative inotropic effects whereas lower amounts of NO elicited positive ones^4^. In addition to stimulating NO-GC, NO has been proposed to alter cardiac function independently of cGMP^5^. Similarly, to the relative confusion about the NO/cGMP-induced cardiac effects, the entity of cardiac cells displaying NO-induced cGMP increases has been a matter of debate. In neonatal cardiac myocytes, FRET-based cGMP measurements yielded NO-induced cGMP signals^6^; in adult rat cardiac myocytes the Fischmeister group detected NO-induced cGMP signals using cyclic nucleotide-gated channels and electrophysiological methods^7^. The Nikolaev group reported that NO evoked small cGMP increases only after prestimulation with a β agonist together with a PDE5 inhibitor as measured using a very sensitive cGMP indicator^8^ (for review see ref. ^9^). Using a similarly sensitive cGMP indicator (PfPKG, cGMP EC_50_ 22 nM), the Andressen group reported undetectable NO-induced cGMP signals in isolated cardiac myocytes^10^. Thus, even using FRET-based cGMP indicators with 10-fold higher cGMP affinity than our one (cGi-500, EC_50_ 500 nM), no global cGMP signals were detectable in cardiac myocytes by other groups, although this does not exclude the existence of highly compartmentalised cGMP. In accordance in our recent report using cGi-500, NO-induced cGMP signals were found to be absent from cardiac myocytes whereas high NO-sensitive cGMP-forming activity was reported to occur in cardiac fibroblasts^11^. Surprisingly, in a co-culture of cardiac fibroblasts and myocytes, cGMP formed in the cardiac fibroblasts entered cardiac myocytes via gap junctions as shown with two groups of gap junction inhibitors (carbenoxolone, connexin 43-mimetic peptides).

It was the aim of the present study to further substantiate the gap junction-mediated transfer of cGMP from cardiac fibroblasts to cardiac myocytes. Here, in mice with specific expression of a FRET-based cGMP indicator in cardiac myocytes (αMHC [α myosin heavy chain]/cGi-500), we demonstrate (I) NO-induced cGMP signals in cardiac myocytes of acute heart slices and (II) dependency of these NO-induced cGMP signals on gap junctions. Furthermore, NO-GC stimulation increased Forskolin (Fsk)- or Isoproterenol (Iso)-stimulated cAMP and phospholamban phosphorylation in heart slices; both being abolished by gap junction inhibitors. Genetic inactivation of NO-GC in Tcf21 (transcription factor 21)-expressing cardiac fibroblasts abrogated the synergistic action of the NO-GC stimulator on Iso-induced phospholamban phosphorylation substantiating the necessity of the cGMP transfer from fibroblasts to myocytes for the observed signals. These data indicate that the fibroblast-derived cGMP inhibits PDE3 in cardiac myocytes thereby increasing cAMP and enhancing downstream effects.

## Results

Recently, we demonstrated the transfer of cGMP from cardiac fibroblasts to myocytes via gap junctions in a primary cell culture model. Obviously, the question whether the cGMP transfer from cardiac fibroblasts to myocytes occurs in native tissue is of high relevance. In order to selectively measure cGMP in cardiac myocytes of native cardiac tissue, we generated mice expressing the FRET-based indicator specifically in cardiac myocytes (αMHC/cGi-500) by crossing mice with a floxed stop cassette in front of the cGMP indicator gene with mice expressing Cre under the control of the αMHC promotor.

**Fig. 1:**
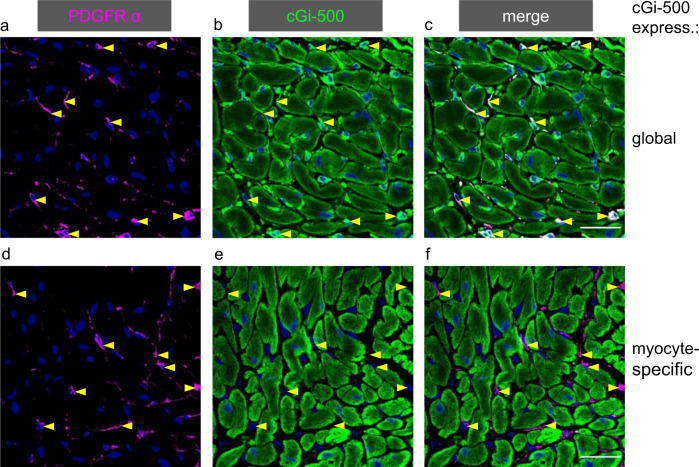
Immunofluorescence staining of cardiac slices demonstrates myocyte-specific expression of the cGMP indicator and the lack of the indicator in fibroblasts of mice with myocyte-specific cGi-500 expression. Fibroblasts stained for PDGFRα (magenta, **a**, **d**) express the cGMP indicator cGi-500 (green, **b**, **e**) only in mice with global (**a**–**c**) but not myocyte-specific (**d**–**f**, αMHC-promoter-driven) expression of the cGMP indicator as shown in the overlay (**c**, **f**, white staining indicates colocalisation). For orientation, yellow arrowheads pointing to fibroblasts are shown. Calibration bar 25 µm.

Immunohistochemical analysis of cardiac slices from the αMHC/cG-i500 mice revealed expression of cGi-500 exclusively in cardiac myocytes because co-localisation of the indicator with fibroblasts – specifically labelled with antibodies against PDGF (platelet-derived growth factor) receptor α – was not apparent (Fig. 1d–f). In contrast, co-labelling of the cGMP indicator and PDGF receptor α signals was clearly observed in mice with global expression of the cGMP indicator (Fig. 1a–c).

### NO-induced cGMP signals occur in cardiac myocytes as measured in living cardiac slices

**Fig. 2:**
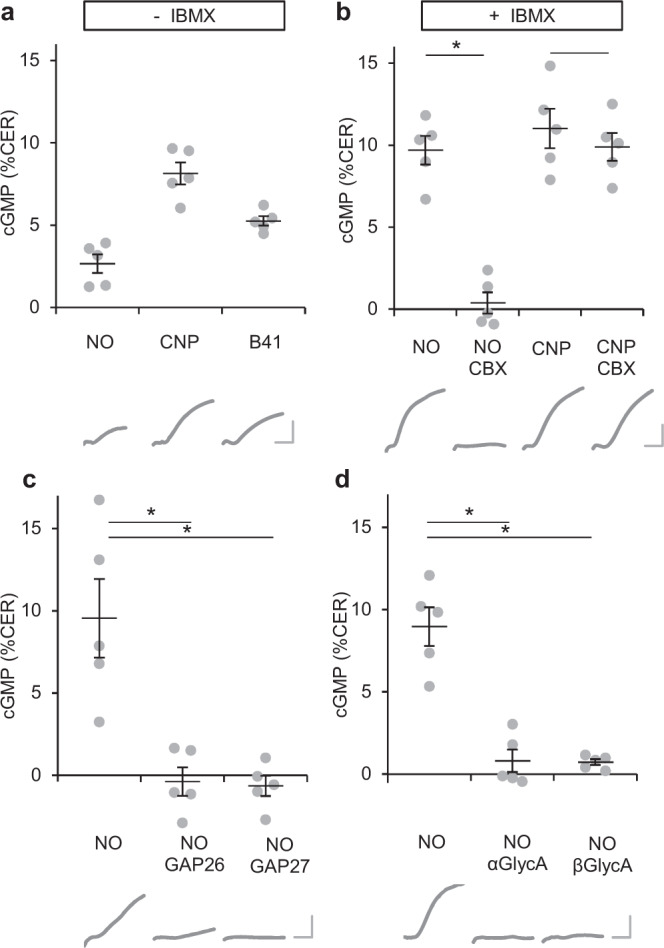
NO-dependent cGMP signals in cardiac myocytes are abolished by a range of gap junction inhibitors. **a** In acute cardiac slices of mice with cardiac myocyte-specific expression of a cGMP indicator, cGMP signals induced by NO (10 µM GSNO), CNP (300 nM) or B41 (10 µM BAY41-2272) were analysed. **b** Preincubation with the gap junction inhibitor carbenoxolone (CBX, 10 µM, 15 min) selectively abolished NO- but not CNP-induced cGMP signals in myocytes (measured in the presence of the broad-spectrum PDE inhibitor IBMX, 500 µM). **c**, **d** Preincubation with (**c**) connexin mimetic peptides GAP26 or GAP27 (25 µM) or (**d**) α- or β-glycyrrhetinic acid (α/β GlycA, 10 µM) inhibited NO-induced cGMP signals in the presence of IBMX. In each panel, individual values obtained with slices from *n* = 5 mice with means ± SEM are shown. *p < 0.05 by Sidak’s multiple comparisons test. Below the diagrams, representative traces obtained under the respective conditions are shown; calibration bar 4% CER (change of emission ratio), 10 min.

To monitor cGMP increases in cardiac myocytes, acute heart slices (250 µm) derived from αMHC/cGi-500 mice were stimulated with the NO donor GSNO (S-nitrosoglutathione), the NO sensitiser/GC stimulator BAY41-2272 or the transmembrane guanylyl cyclase B activator CNP. All stimulators of cGMP synthesis clearly elicited cGMP signals (Fig. 2a). To our knowledge, these are the first FRET-based cGMP measurements in heart slices. The observed increase of the cGMP signals induced by broad-spectrum PDE inhibitor IBMX (isobutyl methyl xanthine) is in support of the notion that our experimental setting allows in fact to determine cGMP signals in cardiac slices (Fig. 2b).

To address the proposed gap junction dependency, slices were preincubated with the gap junction inhibitor carbenoxolone (CBX, 15 min). In accordance with the notion of a gap junction-dependent transfer of NO-stimulated cGMP from fibroblasts to myocytes, CBX abrogated the NO-induced cGMP signals in myocytes whereas the CNP-stimulated cGMP signal remained unaltered (see Fig. 2b). The results were confirmed with mechanistically different inhibitors, connexin 43 mimetic peptides GAP26 and GAP27 (Fig. 2c) as well as α- and β-glycyrrhetinic acid (Fig. 2d) as additional gap junction inhibitors. As far as we know, the results thus demonstrate for the first time in native tissue that cGMP formed upon GSNO incubation in fibroblasts enters cardiac myocytes via gap junctions whereas CNP obviously elicits cGMP signals directly in the myocytes and therefore does not depend on gap junctions.

### siRNA-induced knock down of connexin 43 in fibroblasts prevents NO-induced cGMP in cardiac myocytes

To further substantiate requirement of gap junctions for the cGMP transfer besides the use of inhibitors, a knock out mouse model would ideally be suited. However, knock out of connexin 43 - the dominant connexin isoform responsible for the formation of gap junctions in cardiac ventricle - is lethal shortly after birth^12^. Therefore, we knocked down connexin 43 in a co-culture model of cardiac fibroblasts and cardiac myocytes.

**Fig. 3:**
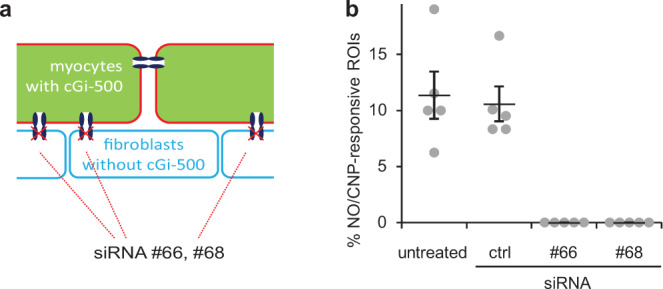
Knock down of connexin 43 in fibroblasts prevents NO-induced cGMP in co-cultured cardiac myocytes. **a** To be able to analyse cGMP specifically in myocytes, cardiac myocytes expressing the cGMP indicator (cGi-500, green) were cultured on cardiac fibroblasts without cGi-500. One day prior to seeding the myocytes, fibroblast’s connexin 43 was knocked down using two different siRNAs (#66, #68). **b** Primary cardiac fibroblasts were transfected with siRNA targeted against connexin 43 (#66, #68) or a non-targeting control (ctrl) siRNA. One day later, cardiac myocytes from knock-in mice expressing a cGMP indicator (cGi-500) were seeded on top. After additional 1–2 days, co-cultures were stimulated for 5 min with NO (10 µM GSNO, 5 min, in the presence of 100 µM IBMX) and cGMP was recorded. Subsequently, viable cells were identified by CNP stimulation (300 nM, 5 min, in the presence of 100 µM IBMX) because CNP stimulates cGMP production directly in myocytes. Presented are NO-responsive cardiac myocytes as a fraction of all viable (CNP-responsive) cardiac myocytes. Shown are individual values obtained with cardiac myocytes from *n* = 5 mice with means ± SEM.

In the model, freshly prepared cardiac myocytes expressing a cGMP indicator are plated directly on cardiac fibroblasts devoid of a cGMP indicator (Fig. 3a). Knock down of connexin 43 in the cardiac fibroblasts was performed by siRNA transfection prior to the co-culture with myocytes. After 2–4 days, cGMP signals are induced with the NO-releasing substance GSNO followed by the addition of CNP (that elicits cGMP signals directly in cardiac myocytes) to identify viable myocytes. A GSNO-induced cGMP signal was observed in ~12% of viable (CNP-responsive) cardiac myocytes; two different siRNAs for connexin 43 blunted the NO-induced cGMP increases whereas a control non-targeting siRNA had no effect compared to the untreated control (Fig. 3b). The results emphasise the requirement of gap junctions for the transfer of cGMP generated in cardiac fibroblasts to myocytes.

### PDE3 is responsible for cGMP degradation in myocytes in cardiac slices

As it is tempting to speculate about NO/cGMP effects in cardiac myocytes, we studied the impact of PDE2 and PDE3 on degradation of NO-induced cGMP. Besides degrading cGMP, these PDEs degrade cAMP and are activated and inhibited by cGMP, respectively, and therefore arise as possible target molecules for cGMP.

**Fig. 4:**
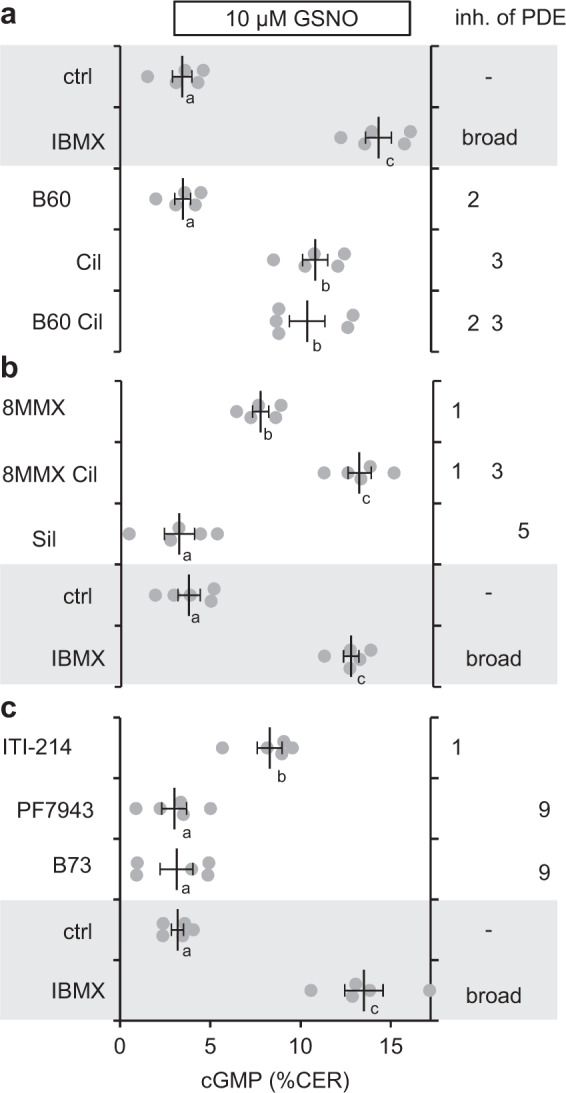
PDE3 is in control of NO-dependent cGMP signals in cardiac myocytes. **a** NO-induced cGMP signals of myocytes in acute cardiac slices were measured in the absence (ctrl) or presence of the broad-spectrum PDE inhibitor IBMX (500 µM), PDE2 (B60, 100 nM BAY60-7550) or PDE3 (Cil, 10 µM cilostamide) inhibitors or a combination thereof. Shown are individual values obtained with slices from n = 5 mice with means ± SEM. ^a, b, c^ means not sharing any letter are significantly different by Tukey’s multiple comparisons test, *p* < 0.05 **b** An analogous experiment was performed with PDE1 (50 µM 8MMX), PDE3 (10 µM Cil) and PDE5 (Sil, 1 µM sildenafil) inhibitors. **c** An analogous experiment was performed with the new PDE1 (5 µM ITI-214) and PDE9 (PF7943, 2 µM PF-0447943; B73, 10 µM BAY73-6691) inhibitors.

To this end, GSNO stimulation of cardiac slices was performed in the presence of the PDE3 inhibitor cilostamide (Cil) or the PDE2 inhibitor BAY60-7550, the broad-spectrum PDE inhibitor IBMX was applied for comparison. Whereas PDE2 inhibition did not affect the cGMP response, inhibition of PDE3 had a marked effect which was almost as high as the one observed for IBMX (Fig. 4a). The results indicate that the majority of NO-induced cGMP generated in fibroblasts entering the myocytes is degraded by PDE3, which is in accordance with the enzyme’s low kM for cGMP. In order to identify the additional PDE isoform involved in the degradation of NO-induced cGMP, cardiac slices were incubated with inhibitors of PDE1 (8MMX, 8-methoxymethyl-3-isobutyl-1-methylxanthine) and PDE5 (Sildenafil), again the broad-spectrum PDE inhibitor IBMX was used for comparison. The results revealed an additional role of PDE1 in degradation of NO-induced cGMP that is apparently smaller than that of PDE3 (Fig. 4b). The combination of the PDE1 and PDE3 inhibitors was as effective as IBMX suggesting that additional PDEs are not involved. In accordance, inhibition of PDE5 had no effect. The cGMP-specific PDE9 is not inhibited by IBMX and has been proposed to be expressed in heart tissue, therefore, two PDE9 inhibitors were tested but found to not have any effect on cGMP degradation in cardiac myocytes (Fig. 4c). Effects of the new inhibitor of PDE1 ITI-214 were also analysed, the obtained result was roughly comparable to the one with 8MMX.

### Fsk- or Iso-induced cAMP levels in cardiac myocytes are enhanced by fibroblast NO-GC-generated cGMP via inhibition of PDE3

**Fig. 5:**
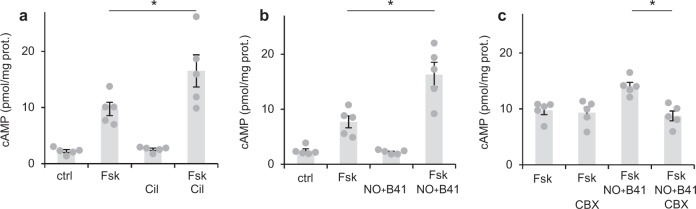
By PDE3 inhibition, NO-induced cGMP enhances forskolin-stimulated cAMP levels. **a** In acute cardiac slices, forskolin (Fsk, 10 µM)-induced cAMP levels as analysed in radioimmunoassays were increased by the PDE3 inhibitor cilostamide (Cil, 10 µM). **b** Similarly, the combination of NO (GSNO, 100 µM) and the NO sensitiser B41 (10 µM BAY41-2272) enhanced forskolin-induced cAMP levels. **c** The gap junction inhibitor CBX (10 µM carbenoxolone) prevented the NO/B41-induced enhancement of Fsk-stimulated cAMP. In each panel, individual values obtained with slices from *n* = 5 mice with means ± SEM are shown. *p < 0.05 by Sidak’s multiple comparisons test.

Thus, we asked whether fibroblast-derived cGMP in cardiac myocytes has an impact on cAMP via PDE3. First, we determined cAMP levels in cardiac slices incubated with the cAMP-increasing forskolin (Fsk) in the absence and presence of the PDE3 inhibitor Cil in radioimmunoassays (Fig. 5a). Compared to non-treated slices (ctrl), Fsk caused a ~4-fold increase of cAMP. The PDE3 inhibitor Cil did not affect cAMP levels in the absence of Fsk, but caused an about 2-fold increase of Fsk-induced cAMP demonstrating that PDE3 participates in degradation of Fsk-stimulated cAMP.

Next, the impact of NO-induced cGMP on cAMP levels was studied. As NO released from GSNO is scavenged very effectively by myoglobin, we combined GSNO with the NO sensitiser/GC stimulator BAY41-2272 that increases NO-GC’s sensitivity towards NO and stimulates NO-independently (Fig. 5b). The combination of GSNO and BAY41-2272 did not increase cAMP levels per se, but caused an enhancement of Fsk-induced cAMP levels. The results indicate that the cGMP formed in response to NO and BAY41-2272 causes inhibition of the PDE3 species in myocytes responsible for cAMP degradation. According to our hypothesis that cGMP in response to GSNO is formed in the fibroblasts and enters myocytes via gap junctions, we analysed whether the gap junction inhibitor CBX abrogates the effect of cGMP on cAMP levels. Whereas preincubation of cardiac slices with CBX (15 min) did not alter cAMP levels induced by Fsk, it clearly abolished the cAMP-increasing effect of GSNO and BAY41-2272 (Fig. 5c). In sum, the data indicate that the cGMP formed in cardiac fibroblasts in response to NO exhibits an inhibitory effect on cAMP degradation of PDE3 in cardiac myocytes, thereby increasing Fsk-induced cAMP.

**Fig. 6:**
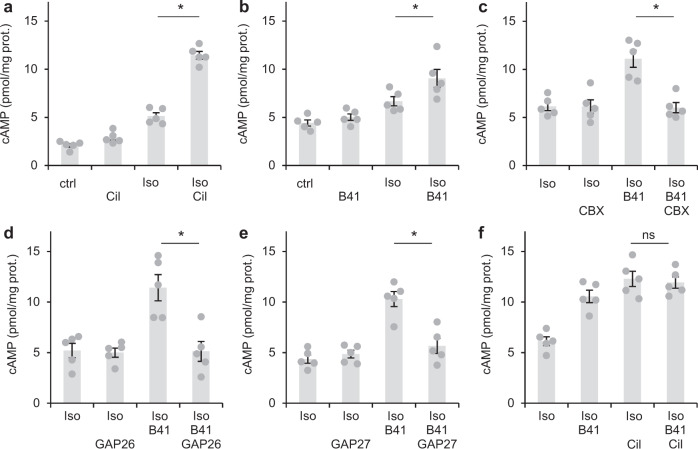
By PDE3 inhibition, NO-induced cGMP enhances Isoproterenol-stimulated cAMP levels. **a** In acute cardiac slices, Isoproterenol (Iso, 1 µM)-induced cAMP levels as analysed in radioimmunoassays were increased by the PDE3 inhibitor cilostamide (Cil, 10 µM). **b** The NO sensitiser B41 (10 µM BAY41-2272) likewise enhanced Iso-induced cAMP levels. **c**–**e** The gap junction inhibitor CBX (10 µM carbenoxolone) and the peptide gap junction inhbitors GAP26 (25 µM) and GAP27 (25 µM) prevented the B41-induced enhancement of Iso-stimulated cAMP. **f** In the presence of the PDE3 inhibitor Cil, the NO sensitiser B41 does not enhance Iso-induced cAMP. In each panel, individual values obtained with slices from *n* = 5 mice with means ± SEM are shown. *p < 0.05 by Sidak’s multiple comparisons test.

Next, we asked whether cGMP produced by NO-GC in fibroblasts likewise increases cAMP induced by the β receptor agonist isoproterenol (Iso). Iso caused an about 2.5-fold increase in cAMP, the addition of the PDE3 inhibitor Cil further increased cAMP levels (Fig. 6a). Does cGMP generated by NO-GC in fibroblasts cause an increase in cAMP in cardiac myocytes? To answer the question, we solely used the NO sensitiser/GC stimulator BAY41-2272 for NO-GC stimulation because NO and Iso inactivate each other^13,14^. As can be seen in Fig. 6b, BAY41-2272 further enhanced the Iso-induced cAMP. Again, we used the gap junction inhibitor CBX to test whether cGMP was formed in cardiac fibroblasts and entered cardiac myocytes where it increased cAMP via PDE3 inhibition (Fig. 6c). As with the Fsk-induced cAMP increases, CBX prevented the effect of the NO sensitiser/NO-GC stimulator on cAMP-levels induced by Iso. To exclude possible non-specific effects of CBX that have been reported, we again used a mechanistically different class of inhibitors, the connexin 43 mimetic peptides GAP26 (Fig. 6d) and GAP27 (Fig. 6e) as gap junction inhibitors. Both inhibitors abolished the increase of cAMP levels induced by the NO sensitiser/NO-GC stimulator clearly arguing against non-specific actions of the gap junction inhibitors. To further substantiate PDE3 as the molecule being targeted by cGMP, we checked whether BAY41-2272 does increase cAMP when PDE3 is already inhibited by the PDE3 inhibitor Cil. The PDE3 inhibitor increased Iso-induced cAMP and BAY41-2272 did not further enhance cAMP in the presence of Iso and Cil (Fig. 6f). The results favour the assumption that the cGMP formed in response to the NO-GC stimulator increases cAMP by inhibiting PDE3.

In sum, the results underpin the conclusion that NO-GC-formed cGMP from cardiac fibroblasts exerts an inhibitory effect on cAMP degradation by PDE3, thereby increasing cAMP in cardiac myocytes.

### Iso-induced cAMP is enhanced by cGMP formed in response to endogenous NO in cardiac slices

**Fig. 7:**
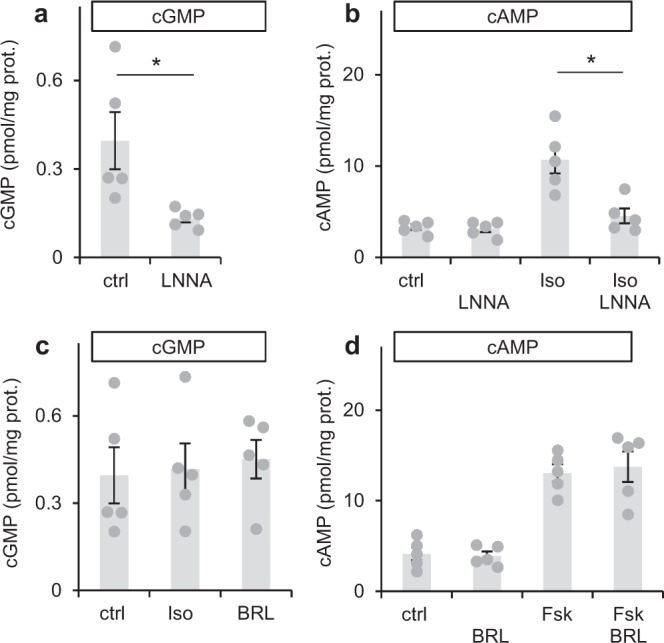
Tonic NO/cGMP production enhances Isoproterenol-induced cAMP levels. **a** In acute cardiac slices, cGMP levels were reduced by the NO synthase inhibitor LNNA (100 µM). **b** Although LNNA did not affect basal cAMP levels, it decreased isoproterenol (Iso, 1 µM)-induced cAMP. **c** The β agonist Iso and the β3 agonist BRL 37344 (100 nM) do not increase cGMP. **d** The β3 agonist BRL 37344 does not increase non-stimulated or forskolin (Fsk, 10 µM)-stimulated cAMP. In panels A + C, B and D, individual values obtained with slices from *n* = 5 mice with means ± SEM are shown. *p < 0.05 by Sidak’s multiple comparisons test.

In order to detect endogenous NO formation that has been reported to occur in the heart under non-stimulated conditions^15,16^, cGMP levels in cardiac slices were analysed in the absence and presence of the NO synthase (NOS) inhibitor LNNA (l-N^G^-Nitroarginine). As can be seen in Fig. 7a, cGMP levels (~0.4 pmol/mg) were reduced by 66% by the NOS-inhibitor indicative of NO-forming activity under non-stimulated conditions.

Does the cGMP formed in response to endogenous NO has an impact on cAMP levels in cardiac slices? Whereas LNNA did not alter cAMP under non-stimulated conditions, the NOS inhibitor almost abolished Iso-induced cAMP levels measured in cardiac slices (Fig. 7b). The results indicate that endogenously produced NO/cGMP exhibits a cAMP increasing, potentially inotropic effect in cardiac slices.

As a freely diffusible gas, NO is capable of crossing cell membranes thereby acting as a paracrine mediator. However, the cells responsible for generation of NO are unclear. Conceivably, NO may be produced in cardiac smooth muscle cells, fibroblasts, endothelial cells or myocytes. Lately, β3-receptors expressed in cardiac myocytes and microvascular endothelial cells were implicated to mediate stimulation of NO/cGMP signalling^17^. In order to test this pathway, the β3 agonist BRL-37344 was used for NOS activation in cardiac slices and the respective cGMP was determined in radioimmunoassays (RIAs). The β3 agonist did not increase cGMP (Fig. 7c) nor did it affect basal or Fsk-stimulated cAMP levels (Fig. 7d) indicating that the β3 agonist does not activate NO/cGMP signalling in cardiac slices under the conditions applied.

### The cAMP/cGMP crosstalk increases phospholamban phosphorylation

**Fig. 8:**
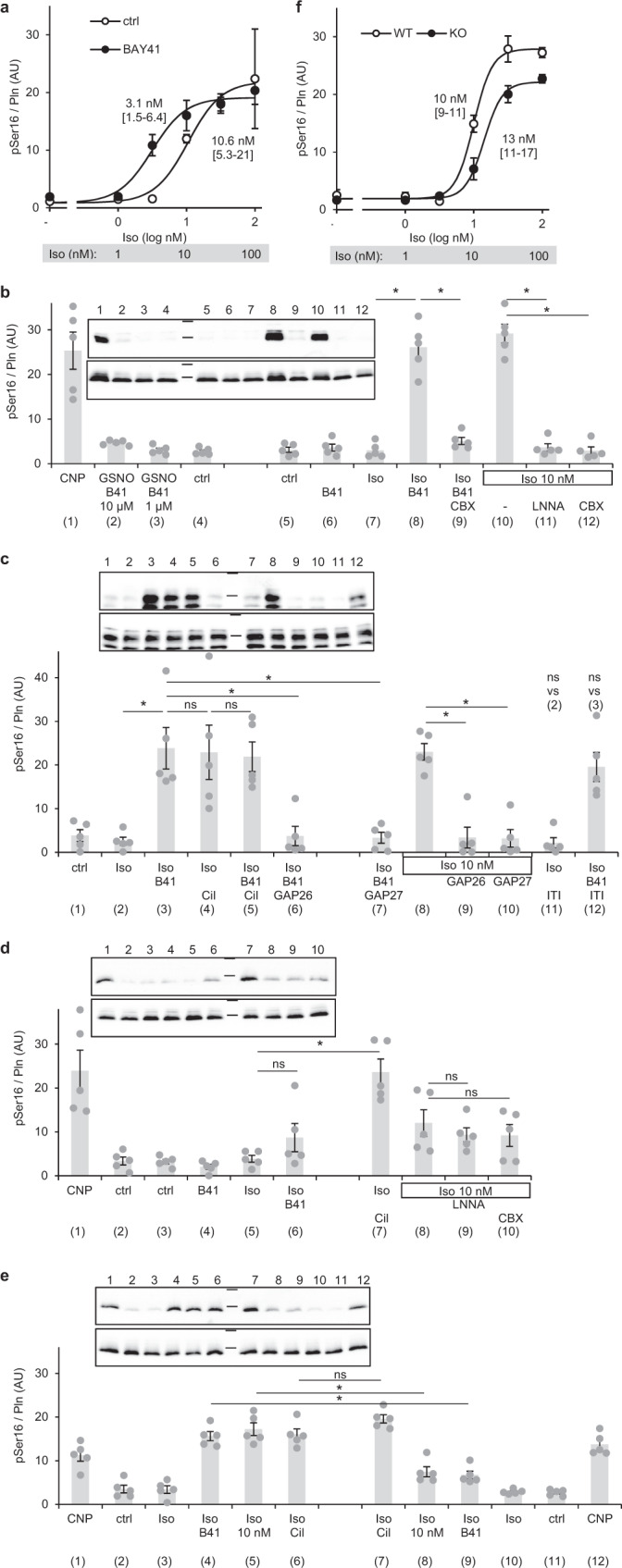
NO/cGMP generated in Tcf21-fibroblasts enhances cAMP-mediated phospholamban phosphorylation via PDE3. **a** The NO-GC stimulator BAY41 (1 µM BAY41‐2272) shifts the concentration response curve of Iso-induced PLN phosphorylation to the left. Shown are means ± SEM obtained with slices from *n* = 5 mice and EC_50_ values with 95% confidence intervals. **b** Lanes 1–4: as published, CNP (1 µM) increases phospholamban phosphorylation (Ser16) in acute acute cardiac slices. In contrast, GSNO (100 µM) with 1 or 10 µM BAY41-2272 have no effect. Lanes 5–9: whereas by themselves, BAY41-2272 (1 µM) and Iso (3 nM) do not increase phospholamban phosphorylation (Ser16), their combination elicits phospholamban phosphorylation in a supra-additive manner, an effect abolished by preincubation with the gap junction inhibitor carbenoxolone (CBX, 100 µM). Lanes 10–12: phospholamban phosphorylation induced by Iso (10 nM) is decreased by preincubation with LNNA (100 µM) or CBX. **c** Lanes 1–5: the PDE3 inhibitor cilostamide (Cil, 1 µM) enhances PLN phosphorylation in the presence of Iso (3 nM) comparable to B41 (1 µM BAY41‐2272); in the presence of Cil, B41 does not further enhance PLN phosphorylation. Lanes 3, 6–10: The peptide gap junction inhibitors GAP26 (25 µM) and GAP27 (25 µM) prevent phosphorylation of PLN induced by Iso (3 nM) and BAY41‐2272 (1 µM) as the ones induced by 10 nM Iso. Lanes 2–3, 11–12: The PDE1 inhibitor ITI-214 (5 µM) does not affect Iso-induced PLN phosphorylation in the absence or presence of B41. **d** In cardiac slices of fibroblast-specific NO-GC1-KO mice (Tcf21-NO-GC1), NO/cGMP-induced enhancement of cAMP‐mediated PLN phosphorylation is abrogated. Whereas CNP (lane 1) and the combination of Iso (3 nM) and the PDE3 inhibitor Cil (1 µM, lane 6) increase PLN phosphorylation, the combination of Iso (3 nM) and BAY41‐2272 (1 µM, lane 6) do not. Lanes 8–10: In the presence of 10 nM Iso, LNNA and CBX do not reduce PLN phosphorylation in the KO. **e** Side by side analysis of Cre-negative littermates of Tcf21-NO-GC1 KOs (left panel vs. right panel) shows greatly reduced effects NO/cGMP on enhancement of cAMP‐mediated PLN phosphorylation (Iso 3 nM ± 1 µM BAY 41-2272 and 10 nM Iso when indicated). Effects of CNP (1 µM) and Iso+Cil (3 nM, 1 µM) are preserved. **b**–**e** In each panel, individual values obtained with slices from *n* = 5 mice with means ± SEM are shown (in **e** *n* = 5 mice of each genotype). *p < 0.05 by Sidak’s multiple comparisons test. Upper blots phospholamban pSer16, lower blots phospholamban, markers 35 and 25 kDa. **f** Fibroblast-specific knock out of NO-GC-1 (Tcf21-NO-GC1) shifts the concentration response curve of Iso-induced PLN phosphorylation to the right compared to Cre-negative littermates (WT). Shown are means ± SEM obtained with slices from *n* = 5 mice of each genotype and EC_50_ values with 95% confidence intervals.

To address the biological relevance of the cGMP-induced cAMP increase in cardiac myocytes, cAMP-dependent phosphorylation of phospholamban (PLN, Ser16) was monitored in western blots. In a systematic approach, we performed an Iso-concentration-response (Fig. 1) in the absence and presence of BAY41-2272; as can be seen in Fig. 8a, the GC stimulator induced a leftward shift of the curve enhancing the pPLN (phosphorylated PLN) signal at 3 and 10 nM Iso.

In the next experiments, a low concentration of Iso (3 nM) was applied that did not increase PLN phosphorylation per se (Fig. 8b, lane 7). The additionally applied NO sensitiser/NO-GC stimulator BAY41-2272 enhanced the cAMP-dependent PLN phosphorylation but did not elicit PLN phosphorylation on its own (Fig. 8b, lanes 6, 8). Thus, the cGMP-dependent cAMP increase translates into a biologically relevant PLN phosphorylation. In accordance with the gap junction-mediated transfer of cGMP from fibroblasts to myocytes, the gap junction inhibitor CBX prevented the NO-GC-dependent increase of Iso-induced pPLN (Fig. 8b, lane 9).

CNP-induced pPLN has been already reported and was observed in our experiental setting. In contrast, maximal NO-GC stimulation did not cause any PLN phosphorylation (lanes 1–3). The results indicate that cGMP signals induced by CNP or NO occur in different compartments of the cardiomyocyte. A higher Iso concentration (10 nM) increased pPLN without additional NO-GC stimulation. In support of the finding that endogenous NO/cGMP increases Iso-induced cAMP, the NOS inhibitor LNNA reduced Iso-induced pPLN as did the gap junction inhibitor (lanes 10–12).

In an additional western blot, the peptide gap junction inhibitors GAP26 and GAP27 are shown to prevent the NO-GC dependent increase of Iso-induced pPLN (Fig. 8c, lanes 6 + 7, 9 + 10). To check whether NO-induced cGMP requires PDE3 to increase Iso-induced pPLN, we tested whether BAY41-2272 can increase pPLN in the presence of the PDE3 inhibitor Cil. Cil and BAY41-2272 increased Iso-induced pPLN to a similar extent (Fig. 8c, lanes 2–4), and the NO-GC stimulator did not further increase Iso-induced pPLN when PDE3 was already inhibited by Cil (lane 5) which is compatible with the assumption that the effect of NO-GC-induced cGMP is brought about by inhibition of PDE3. As the PDE1 inhibitor ITI-214 failed to increase Iso-induced pPLN (Fig. 8c, lanes 11 + 12) we conclude that PDE1 is not involved in degradation of the cAMP pool responsible for the respective phosphorylation under the conditions applied.

In order to verify our findings of a transfer of NO-GC-induced cGMP from fibroblasts to myocytes, we used a completely different approach applying mice (Tcf21-NO-GC1-KO) in which the major NO-GC isoform, NO-GC1, has been specifically deleted in cardiac transcription factor 21-(Tcf21)-expressing fibroblasts. If our hypothesis of a transfer of NO-induced cGMP from fibroblast to cardiac myocytes is right, the NO-induced cGMP effects in cardiac slices on PLN phosphorylation should be vanished or at least be greatly reduced.

The respective Western blot analysis of cardiac slices of the Tcf21-NO-GC1-KO mice revealed that the NO/cGMP-induced enhancement of PLN phosphorylation was greatly reduced (Fig. 8d, lane 6). Pronounced pPLN was still observed with CNP (lane 1) and with Iso only in the additional presence of the PDE3 inhibitor (lane 7). The synergistic action of BAY41-2271 on phosphorylation of PLN induced by 3 nM Iso (lane 6) and even phosphorylation of PLN with 10 nM Iso was just above background (lane 8). In accordance with a deleted NO-GC1, LNNA and CBX did not have an impact on the signal anymore (lanes 9 + 10). To exclude alterations of the mice strain, Iso-dependent pPLN was analysed in WT (wild type) littermates of the Tcf21-NO-GC1-KO mice devoid of the Cre expression in Tcf21 fibroblasts and yielded results undistinguishable for the ones of the WTs used before (Fig. 8e). Comparison of Tcf-21-fibroblast-specific KO mice with WT littermates revealed a rightward shift of the Iso concentration response curve (Fig. 8f) demonstrating enhancement of Iso-induced pPLN by tonic NO and cGMP generation.

In sum, the increase of the cAMP-dependent phospholamban phosphorylation in cardiac myocytes by NO-GC-formed cGMP in cardiac fibroblasts is compatible with the assumption that cGMP supports cAMP’s lusitropic and possibly inotropic action.

## Discussion

In contrast to the crucial role of cAMP in the regulation of cardiac function, the respective effects of cGMP are less clear although numerous in vitro and in vivo reports exist describing NO-cGMP signalling and effects on cardiomyocytes function^18–21^.

To improve the understanding of cardiac effects of the NO/cGMP signalling pathway, we previously monitored the underlying cGMP increases in isolated cardiac cells and found CNP to increase cGMP in cardiac fibroblasts and myocytes whereas NO and other NO-GC stimulators/activators only induced cGMP increases in fibroblasts^11^. Moreover, in a co-culture of cardiac fibroblasts and myocytes, cGMP generated in fibroblasts was able to enter myocytes via gap junctions. The requirement of gap junctions for the transfer of cGMP was demonstrated by the use of two different classes of gap junction inhibitors (carbenoxolone, connexin 43-mimetic peptides).

Still, the concept still demanded demonstration of cGMP transfer in native cardiac tissue.

### Transfer of cGMP formed in fibroblasts to myocytes in cardiac slices

**Fig. 9:**
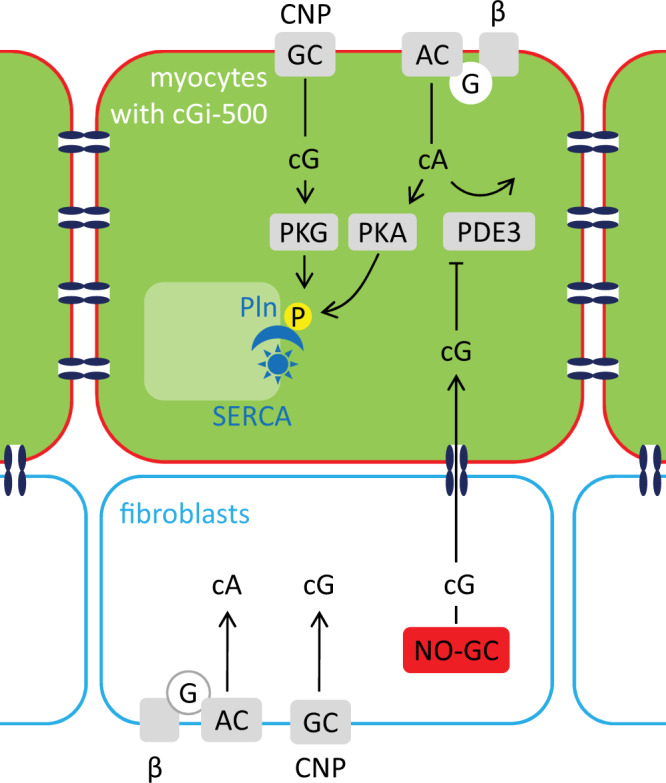
Interplay between NO-GC-generated cGMP from fibroblasts and cardiac myocyte cAMP. cGMP produced by NO-GC specifically in fibroblasts is transferred to cardiac myocytes, inhibits PDE3 and consecutively enhances β adrenoreceptor-induced cAMP signalling and hence phospholamban (Pln) phosphorylation. CNP-induced cGMP and cAMP are generated in both, fibroblasts and myocytes; whether conditions exist leading to concentration gradients required for intercellular transfer is unknown. In myocytes, cGMP generated by CNP-stimulated GC-B elicits Pln phosphorylation via PKG. Apparently, fibroblast-derived cGMP does not reach PKG in relevant concentrations. According to our analyses^11^, PDE3 does not participate in degradation of NO-GC-produced cGMP in fibroblasts.

Accordingly, cGMP measurements were performed in cardiac slices. To detect cGMP specifically in myocytes, cardiac slices were derived from mice (αMHC/cGi-500) that exclusively express the FRET-based cGMP indicator in cardiac myocytes as shown by immunohistochemical analysis. In these acute heart slices, the NO donor GSNO and the natriuretic peptide CNP elicited cGMP signals in FRET measurements. As anticipated for cyclic nucleotides, the signals were increased by the broad-spectrum PDE inhibitor IBMX. To the best of our knowledge, these are the first cGMP signals measured by FRET-based cGMP indicators in intact cardiac slices; so far cGMP signals have only been analysed in isolated cardiac myocytes or fibroblasts. This finding of NO-induced cGMP signals measured specifically in cardiac myocytes together with our previous result of NO-dependent cGMP formation solely occurring in fibroblasts indicated that cGMP must have been transferred via gap junctions^11^. Indeed, different gap junction inhibitors such as CBX, 18α‐ and 18β‐glycyrrhetinic acid and connexin 43-mimetic peptides (GAP26 and GAP27) abrogated the NO-stimulated cGMP signals in native cardiac tissue whereas the cGMP response towards CNP remained unaffected as expected. Thus, the gap junction-dependent transfer of cGMP to cardiac myocytes observed in a cell co-culture model holds true in native cardiac tissue (Fig. 9).

Because the sole use of gap junction inhibitors to address gap junction dependency is regularly criticised, we considered a connexin 43-deficient mouse model lacking the most important connexin isoform of the heart. However, connexin 43-deficiency is lethal^12^. Thus, we applied a co-culture model in which freshly prepared cardiac myocytes are seeded on cardiac fibroblasts cultured before and knocked down the gap junction-forming connexin 43 by siRNA in the fibroblasts before seeding the myocytes. Knock-down clearly abrogated cGMP transfer from fibroblasts to myocytes in the model further demonstrating requirement of the gap-junction forming connexin 43 for transfer of cGMP from fibroblasts to myocytes.

### PDE3 is responsible for cGMP degradation in myocytes in cardiac slices

Analysis of the PDEs responsible for the degradation of fibroblast-derived NO-induced cGMP in cardiac myocytes identified PDE3, the low kM PDE, as the most relevant cGMP-degrading enzyme; inhibition of which had almost the same effect as the broad-spectrum inhibition by IBMX. Thus, fibroblast-derived cGMP must reach a compartment in cardiac myocytes equipped with PDE3. In addition, PDE1 played a role in cGMP degradation, apparently to a somewhat lower extent than PDE3. PDE2, PDE5 and PDE9 did not contribute to degradation of NO-induced cGMP in cardiac myocytes.

In isolated cardiac fibroblasts – in contrast – PDE1 was identified as the major NO/cGMP degrading enzyme and PDE2 had some effect whereas PDE3 did not participate in the reversal of NO-induced cGMP^11^.

### NO-stimulated cGMP increases Fsk- or Iso-induced cAMP levels in cardiac myocytes by inhibition of PDE3

As PDE3, the cGMP-inhibited cAMP-degrading PDE, is the prototype of PDEs proposed to mediate the cGMP/cAMP cross talk, we asked for the impact of fibroblast-derived cGMP on cAMP levels in cardiac myocytes. Indeed, the cAMP levels induced by Fsk in cardiac slices were enhanced by the combination of GSNO plus the NO sensitiser BAY41-2272, the latter known to increase NO-GC’s sensitivity towards NO. The NO donor alone was not sufficient to increase cAMP probably because NO released from GSNO is scavenged very effectively by myoglobin present in high amounts in cardiac myocytes^22^. To answer the question whether the cGMP-dependent increase of Fsk-induced cAMP requires intercellular transfer between fibroblasts and cardiac myocytes, we again applied the gap junction inhibitor CBX and found that preincubation with CBX (15 min) abolished the GSNO and NO sensitiser-dependent enhancement of Fsk-induced cAMP levels.

Similarly, Iso-induced cAMP increases were enhanced by the NO sensitiser BAY41-2272. In these experiments, the NO sensitiser was applied without NO because NO has been published to inactivate Iso^13,14^. The PDE3 inhibitor increased Iso-induced cAMP and in the presence of the PDE3 inhibitor no further enhancement of Iso-induced cAMP by the NO sensitiser BAY41-2272 was seen which is compatible with the notion that PDE3 is the molecule being targeted by cGMP. The cAMP-enhancing effect of BAY41-2272 was prevented by the gap junction inhibitor CBX and the connexin 43-mimetic peptides (GAP26 and GAP27) underlining that cGMP formed in cardiac fibroblasts had to enter cardiac myocytes via gap junctions to increase cAMP via PDE3 inhibition (see Fig. 9).

### Endogenously formed NO supports the cAMP/cGMP crosstalk in cardiac slices

In the early days of NO/cGMP research, the effects of endogenous NO production in the heart were described in numerous reports^15,16,23^. In accordance with those results, the NOS inhibitor LNNA was used to uncover possible effects of tonic NO/cGMP production. Indeed, under NOS-inhibiting conditions, cGMP was decreased by 66% and Iso-induced cAMP levels were reduced to almost basal levels demonstrating that endogenously formed NO/cGMP affects cAMP signals induced by β receptor agonists on the level of PDE3.

In contrast to results that β3-receptors are able to mediate stimulation of NO/cGMP signalling in cardiac cells^17^, the β3 agonist BRL-37344 did neither increase cGMP nor did it affect Fsk-stimulated cAMP levels in cardiac slices (Fig. 8) indicating that the β3 agonist does not activate NO/cGMP signalling at least under the conditions tested.

### PDE3 as a mediator of the cGMP/cAMP crosstalk

In contrast to PDE4, the other major cAMP-degrading PDE of the heart, PDE3 hydrolyses both cAMP and cGMP with relatively high affinities (kM cAMP 0.4 µM; kM cGMP 0.3 µM^24,25^). Yet, PDE3’s 10-fold higher turnover rates for cAMP than for cGMP provide the molecular basis for the cGMP-mediated inhibition of cAMP hydrolysis and coining of the term cGMP-inhibited PDE^26^. The in vivo occurrence of the PDE3-mediated cAMP/cGMP crosstalk first demonstrated in platelets^27^ has also been shown to occur in the heart^28–30^. Here, cGMP elevations were induced by CNP and enhanced noradrenaline-induced contractility in non-failing and failing hearts.

Two isoforms of PDE3 (PDE3A, PDE3B) exist which both occur in cardiac myocytes. The major isoform PDE3A is localised mainly at the sarcoplasmic reticulum^31^ and forms a scaffold with sarcoplasmic reticulum Ca^++^-ATPase (SERCA) thereby controlling the cAMP pool in charge of the contractility relevant phosphorylations (e.g. of phospholamban^32^).

### The cAMP/cGMP crosstalk is paralleled by increased phospholamban phosphorylation

As anticipated, phosphorylation of phospholamban induced by Iso was enhanced by the NO-GC stimulator BAY41-2272 confirming the increase of contractility-relevant cAMP. This effect was dependent on gap junctions because it was abolished by gap junction inhibition with mechanistically different inhibitors, CBX and connexin 43 mimetic peptides GAP26 and GAP27 (see Fig. 9). Furthermore, the NO synthase inhibitor LNNA reduced Iso-induced phospholamban phosphorylation underlining the impact of endogenous NO/cGMP production in cardiac slices. In contrast to CNP that induced phosphorylation of phospholamban when applied alone, high doses of GSNO and the NO-GC sensitiser failed to induce phosphorylation of phospholamban in the absence of a cAMP-increasing agent.

### NO-GC1 in Tcf21-expressing fibroblasts is responsible for the cGMP in the cAMP/cGMP crosstalk

In an attempt to verify our findings of a transfer of NO-GC-induced cGMP from fibroblasts to myocytes and to identify the fibroblasts responsible for NO-induced cGMP formation, we realised Tcf21-lineage tracing emerging as a reliable method to label resident fibroblasts in the heart^33,34^. With the help of the (tamoxifen-inducible) Tcf21-Cre mice and floxed NO-GC1 mice, Tcf21-NO-GC1-KO mice were generated in which the major NO-GC, NO-GC1, is specifically deleted in cardiac fibroblasts.

Indeed, in the cardiac slices of these Tcf21-NO-GC1-KO mice, synergistic action of the NO-sensitiser on phosphorylation of PLN induced by low Iso concentrations and even Iso-induced phosphorylation of PLN per se were greatly reduced. These results I) verify our findings of a transfer of NO-GC-induced cGMP from fibroblast to myocytes and II) identify NO-GC1 in Tcf21-expressing fibroblasts as being responsible for the NO-dependent cGMP in cardiac myocytes enhancing Iso-induced PLN phosphorylation.

In sum, our data demonstrate that NO-GC-generated cGMP originating from Tcf21-positive cardiac fibroblasts apparently by the inhibition of PDE3-mediated cAMP degradation enhances β receptor-induced phospholamban phosphorylation in the non-failing heart. Thus, our data supplies the molecular basis to explain the positive lusitropic and possibly inotropic effects of NO-liberating substances in Langendorff hearts and cardio depressive consequences upon NOS inhibition that have been shown in numerous classical reports^15,16,23,35,36^. Whether and how pathophysiological conditions like heart failure alters NO-induced cGMP effects remains to be elucidated.

## Methods

### Animals

Mice containing a loxP-flanked stop cassette between the CAG promoter and the cGi-500-coding sequence were generated by targeting the Rosa26 locus^11^. Mice expressing the FRET-based cGMP indicator cGi-500^37^ specifically in cardiac myocytes were generated by crossing mice containing a floxed stop cassette in front of the cGi-500-coding DNA sequence with mice expressing Cre recombinase under the control of the αMHC promoter (αMHC-cre, #011038, Jackson^38^).

Generation of the mice containing CAG/stop/cGi-500 was performed with permission of the Danish Ministry for Food, Agriculture, and Fishery and according to the regulations of the Danish Animal Experiments Inspectorate *Dyreforsøgstilsynet* (permission number: 2015-15-0201-00,517). Directive 2010/63/EU of the European Parliament on the protection of animals used for scientific purposes and the German *Tierschutzgesetz* do not consider breeding of genetically altered lines without any harmful phenotype or killing of animals solely for the use of organs or tissues as ‘procedures’ or ‘animal experiments’; consequently, no permission is required. As expected, the introduced genetic alteration causing cardiac myocyte-specific expression of the fluorescent reporter did not result in a harmful phenotype.

Fibroblast-specific KO mice of NO-GC1 were generated by crossing tamoxifen-inducible Tcf21-Cre mice (Tcf21-Cre^39^, kindly provided by Dr. Eric Olson, UT Southwestern) with mice in which NO-GC1 is flanked by loxP sites^40^. For induction of the fibroblast-specific knock out, 8–12 weeks old mice were treated with tamoxifen (1 mg injected intraperitoneally on 5 consecutive days, with permission of the local authority *Landesamt für Natur, Umwelt und Verbraucherschutz Nordrhein-Westfalen* 81‐02.04.2022.A335), and analysed (vide infra) after 3–9 weeks.

Housing, feeding, watering and handling of the animals was performed according to the Directive 2010/63/EU and the German *Tierschutz-Versuchstierverordnung*. Mice were held in a conventional mouse facility (22 °C, 50–60% humidity, 12 h light/dark cycle) with free access to standard rodent chow and tap water. Adult animals (6–12 weeks) of either sex were killed by carbon dioxide and exclusively used for preparation of primary cell cultures and acute cardiac slices.

### siRNA transfection of fibroblasts used in the cardiac fibroblast/myocyte co‐culture model

To obtain cardiac fibroblasts for the co-culture^11^, hearts from WT mice were digested (10 min, 37 °C, 1 mg/ml Collagenase 3 [Worthington 4183] and 160 μg/ml thermolysin [Sigma T7902]), and fibroblasts were plated in fibroblast medium (1% Antibiotic/Antimycotic Gibco #15240, 10 % FBS in DMEM/F-12 Gibco #21331). After approx. 7 days, cells were transfected with two different antiCx43-siRNAs to downregulate Cx43 according to the recommendations of the manufacturer (Silencer select s66666, s66668, Thermo Fisher). A non-targeting siRNA (Silencer select negative control #1, Thermo Fisher) was used as a negative control. One day after transfection, cardiomyocytes were isolated from ubiquitously cGi-500-expressing mice by modified Langendorff perfusion^11^ and plated directly on the transfected cardiac fibroblasts. After additional growth for 1–2 days (2% CO_2_, 37 °C), the co-culture was used for cGMP imaging measurements^11^ on an inverted fluorescence microscope (Zeiss Axiovert 200 with a 10x objective, polychrome V [Till Photonics, Munich], beam splitter [Optical Insights, 505 nm dichroic mirror and 465/30 nm and 535/30 nm bandpass filters] and a CCD camera [Sensicam QE, pco imaging]).

### Preparation of acute cardiac slices

After removal of atria, hearts were transferred to ice-cold Tyrode’s solution without Ca^2+^ (136 mM NaCl, 5.4 mM KCl, 0.33 mM NaH_2_PO_4_, 1 mM MgCl_2_, 5 mM HEPES, 10 mM 2,3-butanedione monoxime (BDM), 10 mM Glucose, pH 7.4) bubbled with carbogen (5% CO_2_, 95% O_2_). The heart (apex upwards) was placed into a custom-made mould subsequently filled with low-melting agarose (4% w/v in Ca^2+^-free Tyrode’s, 37 °C) until the heart was completely covered. After cooling on ice (approx. 10 min), the embedded hearts were cut into 250 µm thick slices on a Vibratome (NVSLM1, World Precision Instruments). After gentle transfer, slices were incubated in ice-cold Ca^2+^-containing Tyrode’s solution (0.9 mM CaCl_2_) bubbled with carbogen for at least 30 min.

### cGMP recording of acute cardiac slices by live cell imaging

Before each measurement, the cardiac slice was incubated in Tyrode’s/Ca^2+^ bubbled with carbogen at 37 °C (15 min), placed on a coverslip mounted on an inverted fluorescence microscope (vide supra) and continuously superfused with Tyrode’s/Ca^2+^ without BDM. Recording was performed^11^ with excitation up to 900 ms (depending on fluorescence intensity) and imaging every 60 s. After 5 min baseline recording, substances as indicated were added to the superfusion solution and recording was performed for additional 30 min. Quantification^11^ was performed using ImageJ^41^ by subtracting a background image (without the slice) from the images obtained during the measurement (with slice) and pixel-wise division of the images of the cyan channel by the images of the yellow channel. To avoid a selection bias, the whole viewing field was selected as region of interest, the average ratio of the whole viewing field was normalised to the average ratio within the last minute of baseline recording and expressed as % change of emission ratio (CER). Ratios during the last minute of stimulation were quantified and statistically analysed.

### Preparation of cardiac cryosections

Acute cardiac slices (1000 µm) prepared as described above were incubated in 5% sucrose (w/v, in Tyrode’s/Ca^2+^, 4 h at 37 °C), transferred to 80% Sucrose (w/v in Tyrode’s/Ca^2+^) and incubated overnight at 37 °C. Subsequently, cardiac sections were embedded in Tissue-Tek O.C.T. compound (Sakura) and stored at −80 °C. Afterwards, the frozen sections were cut into 14 µm sections on a cryostat (Leica CM3050 S), drawn onto adhesion slides (SuperFrost Plus, Fisher Scientific) and stored at −20 °C.

### Histochemical analysis of cardiac cryosections

Sections prepared as described above were thawed, dried at room temperature (RT, 15 min) and fixed with ice-cold 2% paraformaldehyde (w/v, Carl Roth, Karlsruhe, Germany) in PBS (phosphate-buffered saline: 137 mM NaCl, 10 mM Na_2_HPO_4_, 2.7 mM KCl, 1.8 mM KH_2_PO_4_) for 6 min. After washing (3x PBS, 5 min, RT), slices were blocked with 5% (w/v) BSA (bovine serum albumin), 0.75 % Triton X-100 (v/v) in PBS (2 h, RT). Subsequently, slices were incubated with respective antibodies (anti-GFP, ab6556, 1:1000, abcam; anti-PDGF-receptor α, AF1062, 5 µg/ml, R&D-Systems; in PBS, 5 % BSA, 0.025 % Triton X-100; overnight at 4 °C) to detect cGi-500 and identify cardiac fibroblasts^42^, respectively. After washing (3x PBS, 5 min, RT), slices were incubated with secondary antibodies (Alexa488 donkey anti-rabbit, A21206, Thermo Fisher, 1:1000; Alexa594 donkey anti-goat, A11058, Thermo Fisher, 1:1000; in PBS, 5% BSA, 0.025% Triton X-100; 2 h, RT, dark). After washing (3x PBS, 5 min, RT), the slices were covered in aqueous embedding solution (Mount FluorCare DAPI, Carl Roth, Karlsruhe, Germany) and analysed by confocal laser scanning microscopy (Nikon Eclipse Ti-E Inverted Microscope System). Laser and microscope settings were kept identical for different genotypes.

### Measurement of intracellular cAMP or cGMP in radioimmunoassays

For analysis of cAMP or cGMP, acute cardiac slices prepared as described above were incubated in Tyrode’s/Ca^2+^ (30 min, 37 °C). After preincubation (Tyrode’s/Ca^2+^ without BDM) in the absence and presence of CBX as indicated (10 µM, 15 min, 37 °C), slices were incubated with the respective substances as indicated. After 5 min, slices were shock frozen in liquid nitrogen and homogenised in 500 µl ice-cold 70% ethanol using a glass/glass Potter-Elvehjem homogeniser (1000 rpm). After centrifugation (15 min, 4 °C, 21,000 × *g*), cAMP or cGMP in the supernatants was determined by radioimmunoassay^43^. Pellets were resuspended in 1 ml 0.1 M NaOH, 0.1% SDS (sodium dodecyl sulfate) and protein content was determined with BCA Protein Assay (Pierce).

### Western blot analysis of phospholamban phosphorylation

For analysis of phospholamban phosphorylation, acute cardiac slices prepared as described above were incubated in Tyrode’s/Ca^2+^ (30 min, 37 °C), followed by preincubation (15 min) with carbenoxolone (100 µM) or LNNA (100 µM) or vehicle as indicated in Tyrode’s/Ca^2+^ without BDM. Subsequently, slices were stimulated as indicated (5 min, in case of CNP 20 min), snap frozen in liquid nitrogen, homogenised (300 µl; 10 mM Tris, 1 mM EDTA, 1% SDS, pH 8.0, 1 x PhosStop (Roche), 0.5 mM phenylmethylsulfonyl fluoride, 2 µM Pepstatin A, 4 µM benzamidine) with a glass/glass Potter-Elvehjem homogeniser (900 rpm). Debris was removed by centrifugation (15 min, 800 × g, 4 °C), protein content of supernatant was determined with BCA Protein Assay (Pierce) and 4 µg of supernatant protein were subjected to SDS polyacrylamide electrophoresis (15% gels) and Western blotting^44^. Detection was performed using phospholamban pSer16 (A010-12, Badrilla, 1:5000) and phospholamban antibodies (A010-14, Badrilla, 1:5000), anti-rabbit or -mouse IgG (W401B, W402B, 1:3000, Promega), respectively, on gels/blots run in parallel. Chemiluminiscence was measured using SuperSignal West Dura substrate (Pierce) and a CCD camera (GDS 8000 with LabWorks 4.0 software, UVP). Phospholamban pSer16 and phospholamban signals of individual samples were quantified in Labworks, to eliminate intensity variation between blots normalised to mean intensity of specific pSer16 or Pln bands on a membrane, and expressed as pSer16/Pln ratio^44^.

### Statistics and reproducibility

Slices and coverslips were randomly assigned to the test conditions. Data shown are individual values together with means ± SEM (standard error of the mean). Group sizes correspond to the number of animals and were designed to be equal. Statistical analysis was performed using Prism 9 (Graphpad Software, San Diego, CA, USA), *p* < 0.05 was set as threshold for statistical significance.

One-way ANOVA was applied only if variances were not significantly different (Brown-Forsythe’s test) followed by Sidak’s multiple comparisons test (if only one or two pairs of conditions were to be tested) or Tukey’s multiple comparisons test (if all pairs of conditions were to be tested) only if the F test indicated significant differences. Of note, for some measurements specified below (log) the Brown-Forsythe’s test indicated statistically significantly different variances because the variances increased with the means. In these cases, the data were log-transformed and reanalysed; after log transformation, Brown-Forsythe’s test did not indicate significantly different variances, F test indicated significant differences and Sidak’s multiple comparisons test indicated differences between groups as before. For the sake of consistency with the other experiments, the data were therefore depicted in non-transformed form. One-way ANOVA yielded the following *F* statistics:

- Fig. 2b*F*(3,16) = 29.3 *P* < 0.0001, Fig. 2c*F*(2,12) = 14.8 *P* = 0.0006,
- Fig. 2d*F*(2,12) = 35.6 *P* < 0.0001, Fig. 4a*F*(4,20) = 47.5 *P* < 0.0001,
- Fig. 4b*F*(4,20) = 60.4 *P* < 0.0001, Fig. 4c*F*(4,20) = 35.8 *P* < 0.0001,
- Fig. 5a (log) *F*(3,16) = 54.9 *P* < 0.0001, Fig. 5b (log) *F*(3,16) = 53.0 *P* < 0.0001,
- Fig. 5c*F*(3,16) = 8.3 *P* < 0.0001, Fig. 6a*F*(3,16) = 180.7 *P* < 0.0001,
- Fig. 6b*F*(3,16) = 13.2 *P* = 0.0001, Fig. 6c*F*(3,16) = 14.2 *P* < 0.0001,
- Fig. 6d*F*(3,16) = 11.8 *P* = 0.0002, Fig. 6e*F*(3,16) = 20.9 *P* < 0.0001,
- Fig. 6f*F*(3,16) = 22.1 *P* < 0.0001, Fig. 7a+c *F*(3,16) = 3.9 *P* = 0.0282,
- Fig. 7b*F*(3,16) = 16.5 *P* < 0.0001, Fig. 7d*F*(3,16) = 26.3 *P* < 0.0001,
- Fig. 8b (log) *F*(11,48) = 32.5 *P* < 0.0001, Fig. 8c (log) *F*(11,48) = 6.3 *P* < 0.0001,
- Fig. 8d*F*(9,40) = 10.5 *P* < 0.0001, Fig. 8e*F*(11,48) = 38.1 *P* < 0.0001.

For comparison, concentration response curves were fitted with four parameter logistic functions with shared bottom values and hill slopes; whether EC_50_ values differed statistically significantly was analysed by extra-sum of squares F test vs. a shared EC_50_ value (Fig. 8a*F*(1,54) = 6.0 *P* = 0.0179, Fig. 8f*F*(1,54) = 10.9 *P* = 0.0017).

### Reporting summary

Further information on research design is available in the Nature Portfolio Reporting Summary linked to this article.

## Supplementary information

Russwurm_Peer Review File

Supplementary Information FINAL

Description of Additional Supplementary Files

Supplementary Data

Reporting Summary

## Data Availability

All data generated or analysed during this study are included in this published article; source data are available as Supplementary Data and uncropped/unedited blots are available as Supplementary Fig. 1.
