# Supplementary figures and images for: It takes two to tango: cardiac fibroblast-derived NO-induced cGMP enters cardiac myocytes and increases cAMP by inhibiting PDE3
Source: Commun Biol. 2023 May 10;6:504. doi: 10.1038/s42003-023-04880-5 (PMC10172304; doi:10.1038/s42003-023-04880-5)

**Blots of Fig. 8b**

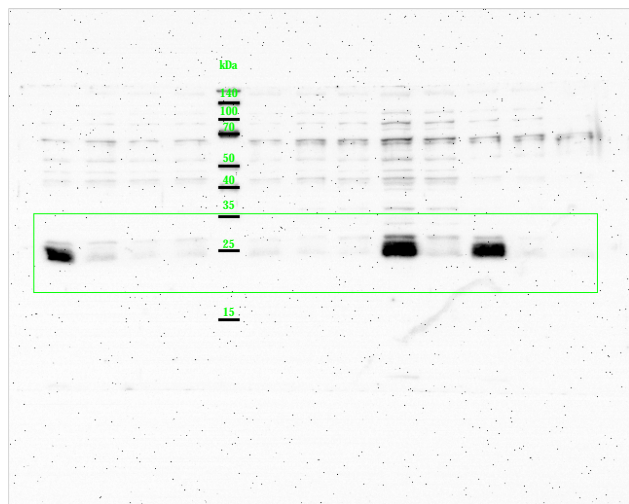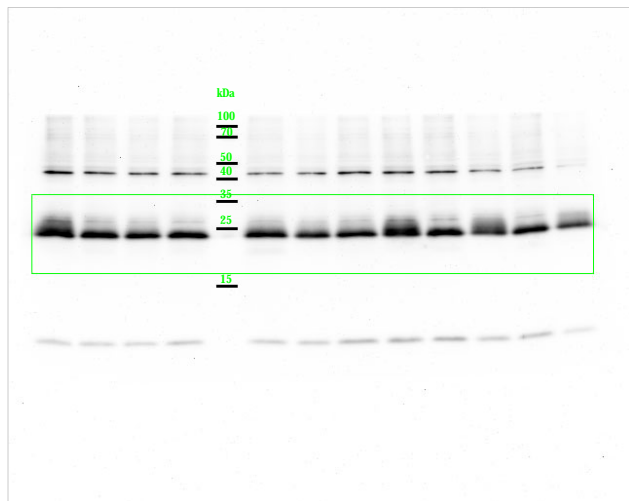

**Blots of Fig. 8c**

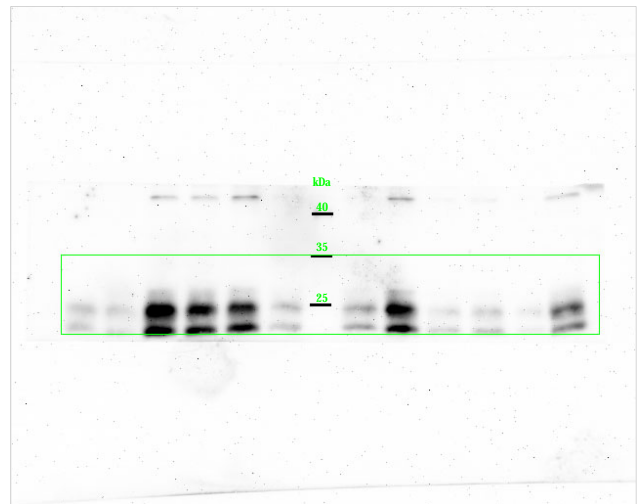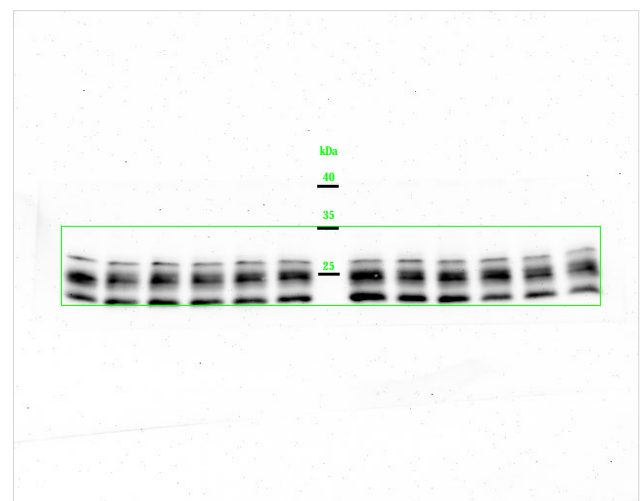

**Blots of Fig. 8d**

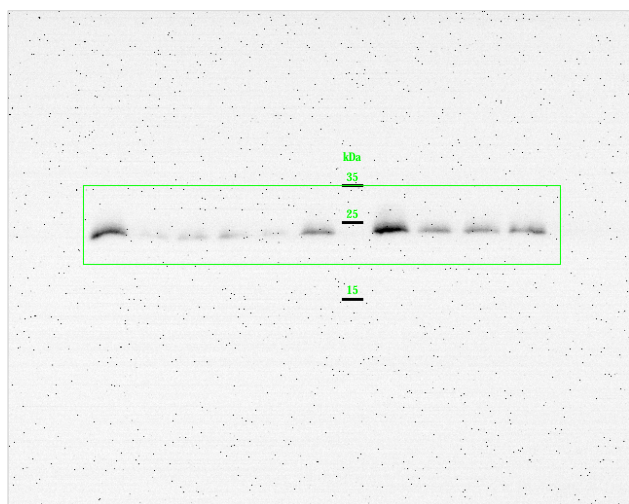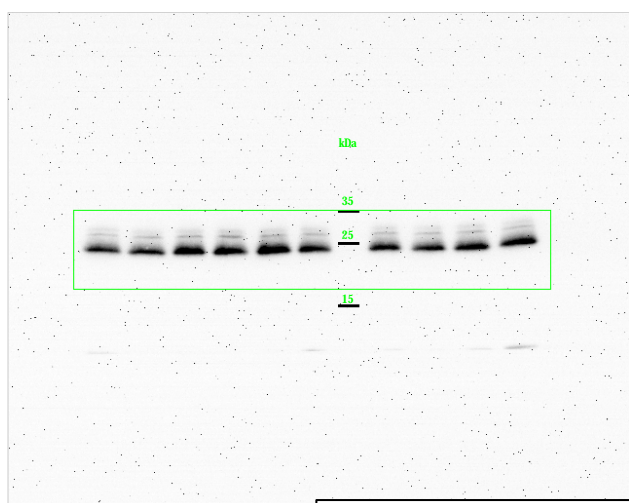

**Blots of Fig. 8e**

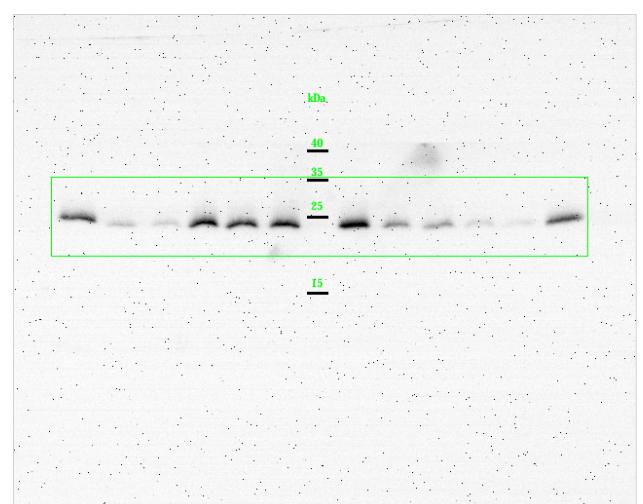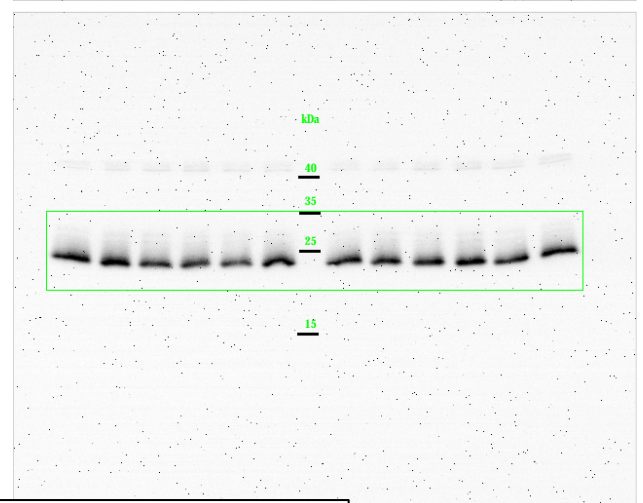

Supplement: Supplementary file 2 — Supplementary Information FINAL [file 42003_2023_4880_MOESM2_ESM.pdf]
