# Supplementary material for: It takes two to tango: cardiac fibroblast-derived NO-induced cGMP enters cardiac myocytes and increases cAMP by inhibiting PDE3
Source: Commun Biol. 2023 May 10;6:504. doi: 10.1038/s42003-023-04880-5 (PMC10172304; doi:10.1038/s42003-023-04880-5)
Supplement: Supplementary file 3 — Description of Additional Supplementary Files [file 42003_2023_4880_MOESM3_ESM.pdf]

Description of Additional Supplementary Files

File name: Supplementary Data

Description: Source data behind the graphs in the paper
