# Supplementary material for: It takes two to tango: cardiac fibroblast-derived NO-induced cGMP enters cardiac myocytes and increases cAMP by inhibiting PDE3
Source: Commun Biol. 2023 May 10;6:504. doi: 10.1038/s42003-023-04880-5 (PMC10172304; doi:10.1038/s42003-023-04880-5)
Supplement: Supplementary file 1 — Russwurm_Peer Review File [file 42003_2023_4880_MOESM1_ESM.pdf]

nature portfolio

Peer Review File

It takes two to tango: Cardiac fibroblast-derived NO-induced cGMP enters cardiac myocytes and increases cAMP by inhibiting PDE3

Open Access This file is licensed under a Creative Commons Attribution 4.0 International License, which permits use, sharing, adaptation, distribution and reproduction in any medium or format, as long as you give appropriate credit to the original author(s) and the source, provide a link to the Creative Commons license, and indicate if changes were made. In the cases where the authors are anonymous, such as is the case for the reports of anonymous peer reviewers, author attribution should be to 'Anonymous Referee' followed by a clear attribution to the source work. The images or other third party material in this file are included in the article's Creative Commons license, unless indicated otherwise in a credit line to the material. If material is not included in the article's Creative Commons license and your intended use is not permitted by statutory regulation or exceeds the permitted use, you will need to obtain permission directly from the copyright holder. To view a copy of this license, visit http://creativecommons.org/licenses/by/4.0/.

Reviewers' comments:

Reviewer #1 (Remarks to the Author):

This is an important contribution highlighting the physiological role of fibroblast to myocyte cGMP transfer in intact mouse Heart tissue. The authors used state of the art biosensor transgenic mouse models, established acute heart tissues slices and performed nice imaging and biochemical experiments which conclude that intercellular cGMP transfer Leads to PDE3 inhibition and increase of myocyte cAMP via cG/cAMP cross-talk. Furthermore, they show that tonic activity of this NO/cGMP/PDE3 pathway blockable by NOS or gap junction inhibitors provide a significant basal inhibition of PDE3 in myocytes, which has an impact also on basal phospholamban phosphorylation in myocytes. I have several points of concern which should be addressed prior to publication:

1. To better characterise the acute slice model, can authors please provide information about cell (myocyte) viability under the conditions used? I understand that paitive controls during imaging such as CNP confirm the only viable cells are measured but what is the picture at the whole tissue level, how many cells can survive?

2. I had some problems with the statistical information. Most of the figure indicate that slices from 5 mice per group were measured. How many slices per mouse? I understood that data from individual slices of the same mouse were averaged before stat analysis. Why not showing every slice data point aus use nested ANOVA analysis available in Prism? Was the analysis for normal distribution performed alltogether before applying parametric tests?

3. I was surprised to see such a dramatic effect of NOS inhibitor and CBX on Iso induced cAMP and pPLN suggesting that almost the whole pPLN at 10 nM iso comes from intercellular NO transfer and pde3 inhibition, leaving virtually no additional role for direct cAMP stimulation in myocytes. To unequivocally demonstrate this fact I would suggest some important controls which I was missing here:

a. Do the peptide gap junction inhibitors have the same effect as CBX?
b. Can the authors please use also co-Culture model with and without Cx43 knockdown in fibroblasts to probe for 1. pPLN under iso stimulation plus minus NOS inhibitor. 2. effect of cilostamide on pPLN.
c. Compare myocytes alone vs co-culture in terms of iso (e.g. 3 and 10 nM) effects on pPLN. It is clear that both should stimulate pPLN in myocytes alone but not in co-culture
d. Compare basal PDE3 activity (preferably by biochemical activity assay) in co-culture (with and without Cx43 knockdown) and myocytes alone.
e. Domonstrate that in myocytes alone CBX and NOS inhibitor do not reduce iso stimulated cAMP

4. the authors are often talking about inotropic effects. While contractility itself was not measured, pPLN is a surrogate marker rather for lusitropic than initropic effect. How about looking at calcium transients (by epifluorescence fura2 or fluo4 imaging) in acute slices? Normally they should be electrically pacable for calcium transient recordings, while cell shorting is rather unrealistic to measure

Reviewer #2 (Remarks to the Author):

In this nice ex vivo studies of freshly prepared murine cardiac slices, Menges et al. extended their previous studies in co-cultured cardiac myocytes and fibroblasts which indicated that NO-induced cGMP formed in cardiac fibroblasts enters cardiac myocytes via gap junctions thereby turning cGMP into an intercellular signalling molecule (Ref. 11 of the present manuscript). Here the authors

prepared ventricular slices from mice with cardiomyocyte-specific expression of a cGMP-FRET sensor. They show that application of NO increases cGMP levels in cardiomyocytes. These responses were abolished by connexin (Cx) 43 inhibitors, suggesting a Cx-43-dependent transfer of cGMP from non-myocytes (probably fibroblasts) to myocytes. Furthermore the present studies confirm that cGMP inhibits the dual substrate esterase PDE3A and thereby augments the cAMP-responses induced by isoprenaline. Ultimately this improves cAMP/PKA-dependent phospholamban phosphorylation. To corroborate such experiments in cardiac slices, the authors performed experiments in co-cultured Cx-43-deficient cardiac fibroblasts and cardiomyocytes. These experiments also indicated that the NO-dependent increases of cGMP in cardiomyocytes require the presence of Cx-43, suggesting that NO stimulates soluble guanylyl cyclase-dependent cGMP production in non-myocytes and transfer of cGMP to neighboring cardiomyocytes via Cx43. If these observations are relevant "in vivo", this would translate in a beneficial fibroblast-to-myocyte cGMP-mediated communication.
The study is nicely designed, the experiments are well conducted and the manuscript is very clearly written and easy to follow. The results are presented in comprehensive way, except Figure 4. This figure is not understandable and the authors may consider to present these data in a different way.
Major concerns:
1. As stated in the intro section (page 3, lines 20-30), the published in vitro studies about NO-GC-cGMP signalling in cultured or freshly isolated adult cardiomyocytes led to discrepant results about NO-cGMP signalling. Some authors detected NO-induced cGMP signals in cardiomyocytes, whereas others (including Menges et al) did not detect increased cGMP levels in cardiomyocytes exposed to NO. The reason for such discrepancies are unclear. The buffers and gas systems used may play a role and either preserve or inactivate NO before sufficient amounts enter the cardiomyocyte. The authors do not mention that several in vivo studies concur in the notion that NO or sGC activators indeed directly increase cGMP levels in cardiomyocytes. Some of these studies are referred below. Hence, studies in vitro or ex vivo may represent an artificial situation. Such in vivo studies and also the experimental conditions potentially limiting NO bioavailability in cultured myocytes and cardiac slices have to be carefully discussed.
Some references showing NO-cGMP signalling in cardiomyocytes in vitro and in vivo:
- Kolijn, D., et al (Frontiers in Physiology, 11(345), 2020) investigated the effect of activation of soluble guanylyl cyclase (sGC) on cardiomyocyte function in a hypertensive animal model with diastolic dysfunction and in biopsies from human heart failure with preserved ejection fraction (HFpEF). sGC activation improved cardiomyocyte function, reduced inflammation and oxidative stress. Moreover, immunohistochemistry and electron microscopy revealed the presence of NO-GC in cardiac myocyte and a translocation of sGC protein towards the intercalated disc and t-tubuli under disese conditions.
- Paulus WJ and Bronzwaer JG. (Am J Physiol Heart Circ Physiol. 287(1):H8-13, 2004) review that in isolated cardiomyocytes, administration of NO increases cGMP-induced phosphorylation of troponin I, which prevents calcium-independent diastolic cross-bridge cycling and concomitant diastolic stiffening of the myocardium.
- Lee DI et al (Nature. 519(7544):472-6, 2015) measured intracellular cGMP generation in adult myocytes expressing the cGMP-fluorescent sensor, FlincG and showed cGMP stimulation by DEANO.
- Frankenreiter S, et al (Cardiovasc Res. 114(6):822-829, 2018) evaluated ischemia/reperfusion injury in mice with a cardiomyocyte-specific deletion of NO-GC (CM NO-GC KO) and in control siblings (CM NO-GC CTR) subjected to an in vivo model of AMI. Their studies demonstrated an important role of CM NO-GC for the cardioprotective signalling following AMI in vivo. The authors concluded that CM NO-GC function is essential for the beneficial effects on infarct size elicited by pharmacological elevation of cGMP

2. Menges et al must discuss that the drugs which they used to block Cx-43 are not specific. Hence, besides blockade of Cx 43, additional effects may have contributed to the inhibition of the cGMP response in cardiomyocytes.

In particular:
Carbenoxolone is a synthetic derivative of glycyrrhizic acid (from licorice root) which has many different actions: mineralocorticoid activity, stimulation of prostaglandin production, inhibitory effect on cell differentiation. Moreover it inhibits the conversion of inactive cortisone to cortisol by blocking 11β-hydroxysteroid dehydrogenase (11β-HSD). Carbenoxolone is a modestly potent, reasonably effective, water-soluble blocker of gap junctions
Gap 26 is a peptide, corresponding to residues 63 - 75 of connexin 43, which is a gap junction blocker. But it also inhibits IP3-induced ATP release, without inhibiting gap junctional coupling in endothelial cells.
etc.
3. To overcome these pharmacological limitations, the authors used siRNA to downregulate Cx-43 in cultured cardiac fibroblasts and then co-cultured such cells with freshly isolated adult murine cardiomyocytes during 1-2 days. This experiment raises at least two questions: how efficient wass the generation of fibroblast-cardiomyocyte gap junctions in this short time? How many adult murine cardiomyocytes remained viable after 1-2 days? This is not described in the manuscript. But the facts that the authors report the cGMP responses in "percent of CNP-responsive cells" and that overall from such CNP-responsive cells only 10% were responsive to NO, suggests that most of the cardiomyocytes were not viable. Could the content of the broken cardiomyocytes affect the NO/cGMP responses?
4. How relevant is this fibroblast-cardiomyocyte crosstalk "in vivo"? As illustrates in Figure 1 of the manuscript, a healthy adult heart contains few fibroblasts (this figure illustrates about 10 fibroblasts scattered between 50 cardiomyocytes). Not only the "number" but also the "contact area" might be a problem. It seems that even for the few "big" cardiomyocytes which are in direct contact with a "small" fibroblast, the area of direct cell-cell contact is very small. If NO-derived cGMP only difuses through this small space, it may not reach functional levels or targets within the big cardiomyocyte (in vivo).
5. I wonder whether the authors could validate their ex vivo data with an in vivo approach, for instance using mice with a fibroblast-restricted deletion of NO-GC (floxed NO-GC mice were previously generated in their group and are possibly available for such studies)

Reviewer #3 (Remarks to the Author):

Menges et al have earlier shown that cGMP produced by the NO-GC in fibroblasts is transferred to cardiomyocytes through gap junctions. The novelty to this study is that they show this in intact cardiac tissue, that the cGMP levels are regulated by PDE1 and PDE3 and that the cGMP produced in fibroblasts also affects cAMP levels and phospholamban phosphorylation in cardiomyoycytes. The manuscript is a nice contribution to explain the unresolved issues about NO and cGMP in cardiomyocytes and provide more information on compartmentation and cross-talk between cGMP and cAMP. It is an elegantly performed study and nicely written, however I have some concerns.

Major concerns:
1. The conclusion that cGMP from fibroblasts increases cAMP levels through PDE3 inhibition is a bit bold based on the evidence the authors provide in the manuscript. This conclusion is clearly stated in the title and throughout the text. I agree that this is the most likely explanation but the results shown are only indirect evidence. Even though PDE3 regulates the cGMP levels in cardiomyocytes after NO-GC activation in fibroblasts, and both NO-GC activation and PDE3 inhibition increase cAMP levels after FSK/Iso stimulation independently, these are only indirect evidences that the effect of NO-GC on cAMP is through PDE3. Thus, the authors should do the same experiments comparing cAMP levels and PLB-phosphorylation after GSNO/B41 stimulation in presence of Cil+FSK/Iso (vs Cil+FSK/Iso without GSNO/B41 and vs FSK/Iso with GSNO/B41). If GSNO does not give a further increase in cAMP than

FSK/Iso+Cil, this will provide better evidence that PDE3 is involved. If GSNO/B41 gives an increase above PDE3 inhibition, this indicates that other mechanisms could be involved.
2. The authors conclude that there are no other PDEs involved than PDE1 and PDE3 as IBMX give the same cGMP levels as the combined inhibition of PDE1 and PDE3 inhibition. PDE9 has also been shown to be expressed in cardiomyocytes and should at least been discussed. IBMX do not inhibit PDE9, thus the authors cannot rule out that PDE9 could also regulate the NO-GC-induced cGMP.
3. PDE1 is also a dual PDE, thus even though PDE1 has not been shown to be involved in cross-talk between cGMP and cAMP(to my knowledge) it should be considered.
4. 12% is a low number of cardiomyocytes that responds to GSNO (figure 3)- why is there so few cells that responds, are there not gap junctions between all fibroblasts and cardiomyocytes? This should at least be commented on. Will it be possible to visually show that there are not gap junctions between all cells or is it that GSNO do not increase cGMP in all fibroblasts?

Minor:
5. Spell out abbreviations when first mentioned: cAMP, cGMP, CER…
6. mGC – not a common name for the membrane bound GCs. A more common name is the particulate GC (pGC)
7. NO-GC – not the most common name for the soluble GC. Maybe use sGC instead?
8. More details on incubation/stimulation times should be provided either in the methods or figures.
9. What is the definition of CER (figure 2 and 4)?
10. Figure 3B. The fraction of how many cells that responded to NO is shown from 5 experiments. How many cells in each experiment and condition? This is important information if there are fewer cells measured in those with siRNA.
11. Figure 4. I agree that IBMX is broad, but maybe specify which PDEs that are inhibited as not all PDEs are?
12. The concentration of cilostamide used is higher than needed and recommended based on selectivity. 1 µM should be enough. PDE2 and 5 will also be affected at 10 µM according to Sudo et al, Biochemical Pharmacology, 2000. This would probably not change the results in this study as PDE2 and PDE5 inhibition did not do much, however it should be taken into consideration.

Reviewer I

1. To better characterise the acute slice model, can authors please provide information about cell (myocyte) viability under the conditions used? I understand that paitive controls during imaging such as CNP confirm the only viable cells are measured but what is the picture at the whole tissue level, how many cells can survive?

Ad 1) Acute cardiac slices used in the FRET measurements were used shortly after preparation and incubation (30 min – 4 hours in the presence of carbogen). We obtained robust reproducible signals with CNP and NO-GC activators. The signals were markedly increased by various PDE inhibitors which - in our opinion - verifies the signals as cyclic nucleotides.

Also, analysis of slices by cAMP-RIAs and PLN-Western-Blots produced reproducible signals after Iso and Forskolin stimulation arguing against uncontrollable dying of the slices.

A recent publication states "Thin heart tissue slices (300 µm) have since been shown to achieve high viability, retain typical tissue architecture, contractility, calcium handling, and conduction velocity for 24 h ". They "do not require external perfusion, and obtain oxygen and nutrients directly from culture media". (Meki et al. (2021) Heart Slices to Model Cardiac Physiology. Front Pharmacol 12:617922 https://pubmed.gov/33613292)

Also, another article states preserved function and structure of rat myocardial slices for 24 h (Pitoulis et al. (2020) Myocardial slices come to age: an intermediate complexity in vitro cardiac model for translational research Cardiovasc Res. 116:1275-1287 https://pubmed.gov/31868875)

2. I had some problems with the statistical information. Most of the figure indicate that slices from 5 mice per group were measured. How many slices per mouse? I understood that data from individual slices of the same mouse were averaged before stat analysis. Why not showing every slice data point aus use nested ANOVA analysis available in Prism? Was the analysis for normal distribution performed alltogether before applying parametric tests?

In most cases (FRET measurements, PLN Western blots), slices from 1 mouse were measured under all conditions given in the respective figures and the whole experiments was repeated with n=5 mice. Thus, for every condition, n=5 slices from n=5 mice were used.

Specifically, because of technical reasons we are only able to measure 4-5 slices obtained from 1 mouse in FRET measurements per day. These 4-5 slices are allocated to 4 (or 5) different conditions as shown in the Figs. For PLN Western blots, up to 12 conditions were measured

with slices from one mouse; again, for every condition depicted n=5 slices from n=5 mice were used. For RIA measurements, 3 slices per condition were used and averaged (=technical replicates) and averaged values from n=5 mice are depicted. Although nested ANOVA can be calculated, it is difficult to depict, i.e. to clarify which values were obtained from one mouse. For the sake of consistency across figures, we thus depicted the single values (means of 3 slices) of n=5 mice.

Because the power to detect deviations from normal distribution with n=5 values is low and indicated no significant deviation from normality, we checked normality of residuals and homogeneity of variances. Only in rare cases, these tests indicated deviations from these assumptions because variances increased with the means. In these cases, these data were log-transformed and reanalysed. Thereafter, variances were not significantly different but ANOVA and post hoc tests indicated differences between the groups as before transformation. For the sake of consistency across figures, the data were therefore depicted in non-transformed form.

3. I was surprised to see such a dramatic effect of NOS inhibitor and CBX on Iso induced cAMP and pPLN suggesting that almost the whole pPLN at 10 nM iso comes from intercellular NO transfer and pde3 inhibition, leaving virtually no additional role for direct cAMP stimulation in myocytes.

Ad 3) Sorry, we never meant to say that Iso-induced cAMP does not play a role in PLN phosphorylation. One should keep in mind that NO-GC-induced cGMP alone does not induce any PLN phosphorylation by itself. The particular conditions of the Western Blot were chosen to illustrate the effect of NO-induced cGMP on Iso-induced cAMP. We now included an Iso-concentration-response (new Fig. 8A; 0; 1; 3; 10; 30; 100 nM Iso) in the absence and presence of BAY41-2272 showing that the GC stimulator induces a leftward shift of the curve strongly enhancing the pPLN signal at 3 and 10 nM Iso. The result is described from page 9 line 28 to page 10 line 3.

Our results indeed demonstrate that in the presence of low Iso concentrations NO-induced cGMP apparently exerts a pronounced impact on pPLN. In the literature, functional cardiac responses are usually provoked by Iso concentration between 1- 10 nM.

To unequivocally demonstrate this fact I would suggest some important controls which I was missing here:

a. Do the peptide gap junction inhibitors have the same effect as CBX?

Ad 3a) We now present a Western Blot of WT cardiac slices showing that the peptide gap junctions inhibitors prevent the enhanced phosphorylation of PLN induced by 3 nM Iso and BAY41-2272 in cardiac slices as did CBX (new Fig. 8C lanes 6+7 vs lane 3). In addition, the peptide inhibitors did abolish PLN phosphorylation induced by 10 nM Iso as did CBX (lanes 8-10).

Moreover, also the enhancement of Iso-induced cAMP by BAY41-2272 measured in RIAs was prevented by the peptide gap junction inhibitors in cardiac slices (new Fig. 6 D+E).

The results are described on page 10 line 19-21 (pPLN Western blot) and page 8 line 21-25 (cAMP RIA).

b. Can the authors please use also co-Culture model with and without Cx43 knockdown in fibroblasts to probe for 1. pPLN under iso stimulation plus minus NOS inhibitor. 2. effect of cilostamide on pPLN.

Ad 3b) First, we have to clarify that we never observed endogenous NO-forming activity in cell culture but only in acute aortic rings, brain or now in cardiac slices. Accordingly, in the co-culture, we do not expect an effect of the NOS inhibitor. Additionally, it is almost impossible to generate sufficient material for Western blotting in the co-culture model. Relevant in the co-culture are the myocytes in which phosphorylation of PLN should be monitored. To avoid the use of very many animals for the cell culture, we used another experimental approach described below to satisfy the referee's demands.

To be able to identify the fibroblasts responsible for NO-induced cGMP formation, we came across a study showing that resident fibroblasts in the heart can be uniformly labelled as transcription factor 21-(Tcf21)-expressing cells. With the help of mouse line with a tamoxifen-inducible Cre under control of the Tcf21 promoter (Tcf21-Cre) and a mouse line in which an exon of the alpha1 subunit of heterodimeric NO-GC1 is floxed, we generated mice in which the major NO-GC, NO-GC1, is specifically deleted in cardiac fibroblasts (Tcf21-NO-GC1-KO). Therefore, if our hypothesis of a transfer of NO-induced cGMP from fibroblast to cardiac myocytes is right, the NO-induced cGMP effects in cardiac slice on PLN phosphorylation should be vanished or at least be greatly reduced.

Western blot analysis of cardiac slices of the Tcf21-NO-GC1 KO mice revealed that the NO/cGMP-induced enhancement of Iso-induced PLN phosphorylation is greatly reduced (new Fig. 8D). Pronounced pPLN is still observed with CNP (lane 1) and with 3 nM Iso only in the additional presence of the PDE3 inhibitor (lane 7 vs lanes 5 and 2-3). The synergistic action of

BAY41-2271 on phosphorylation of PLN induced by 3 nM Iso (lane 6) and even phosphorylation of PLN with 10 nM Iso (lane 8) is just above background. In accordance with a deleted NO-GC1, L-NNA does not have an impact on the signal anymore (lane 9 vs lane 8).

The results are described on page 11 lines 7-13.

c. Compare myocytes alone vs co-culture in terms of iso (e.g. 3 and 10 nM) effects on pPLN. It is clear that both should stimulate pPLN in myocytes alone but not in co-culture

Ad 3c) We consider the Tcf21-NO-GC1 KO mice in which NO-GC1 is specifically deleted in Tcf21-expressing fibroblasts as a model for myocytes not receiving any cGMP and present the results of the Western Blot (discussed above, Fig. 8D) that the pPLN induced by 10 nM Iso and the synergistic action of the NO-GC activator BAY41-2272 on Iso-induced PLN phosphorylation are greatly reduced.

d. Compare basal PDE3 activity (preferably by biochemical activity assay) in co-culture (with and without Cx43 knockdown) and myocytes alone.

Ad 3d) Unfortunately, PDE3 activity within a cell cannot be measured. As the respective measurements can only performed in broken cell preparations, PDE3 activity in a given tissue solely depends on the added cyclic nucleotide concentrations and possibly on so far unknown covalent modification.

e. Domonstrate that in myocytes alone CBX and NOS inhibitor do not reduce iso stimulated cAMP

Ad 3e)

Again, we considered the Tcf21-NO-GC1 KO mice in which NO-GC1 is specifically deleted in Tcf21-expressing fibroblasts as a model for myocytes not receiving any cGMP. In Western blots, we demonstrate that in cardiac slices of Tcf21-GC1-KOs, phosphorylation of PLN induced by 10 nM Iso is greatly decreased and the reduction by CBX and NOS inhibitor is lost accordingly. (Fig. 8D, lanes 8-10). The results are given on page 11 line 12-13. Due to the high amount of newly produced data added already to the ms., the Iso-induced cAMP measurements were omitted.

4. the authors are often talking about inotropic effects. While contractility itself was not measured, pPLN is a surrogate marker rather for lusitropic than initropic effect. How about looking at calcium transients (by epifluorescence fura2 or fluo4 imaging) in acute slices?

Normally they should be electrically pacable for calcium transient recordings, while cell shorting is rather unrealistic to measure

Ad 4) Again, we would like to perform the suggested experiment but, in this case, have to admit that we are technically not able to perform the suggested experiments. Instead, the statements about the ionotropic cGMP effects were attenuated (page 11 line 22, page 16 line 12).

Reviewer II

1. As stated in the intro section (page 3, lines 20-30), the published in vitro studies about NO-GC-cGMP signalling in cultured or freshly isolated adult cardiomyocytes led to discrepant results about NO-cGMP signalling. Some authors detected NO-induced cGMP signals in cardiomyocytes, whereas others (including Menges et al) did not detect increased cGMP levels in cardiomyocytes exposed to NO. The reason for such discrepancies are unclear. The buffers and gas systems used may play a role and either preserve or inactivate NO before sufficient amounts enter the cardiomyocyte. The authors do not mention that several in vivo studies concur in the notion that NO or sGC activators indeed directly increase cGMP levels in cardiomyocytes. Some of these studies are referred below. Hence, studies in vitro or ex vivo may represent an artificial situation. Such in vivo studies and also the experimental conditions potentially limiting NO bioavailability in cultured myocytes and cardiac slices have to be carefully discussed.

Some references showing NO-cGMP signalling in cardiomyocytes in vitro and in vivo:

- Kolijn, D., et al (Frontiers in Physiology, 11(345), 2020) investigated the effect of activation of soluble guanylyl cyclase (sGC) on cardiomyocyte function in a hypertensive animal model with diastolic dysfunction and in biopsies from human heart failure with preserved ejection fraction (HFpEF). sGC activation improved cardiomyocyte function, reduced inflammation and oxidative stress. Moreover, immunohistochemistry and electron microscopy revealed the presence of NO-GC in cardiac myocyte and a translocation of sGC protein towards the intercalated disc and t-tubuli under disese conditions.

- Paulus WJ and Bronzwaer JG. (Am J Physiol Heart Circ Physiol. 287(1):H8-13, 2004) review that in isolated cardiomyocytes, administration of NO increases cGMP-induced phosphorylation of troponin I, which prevents calcium-independent diastolic cross-bridge cycling and concomitant diastolic stiffening of the myocardium.

- Lee DI et al (Nature. 519(7544):472-6, 2015) measured intracellular cGMP generation in adult myocytes expressing the cGMP-fluorescent sensor, FlincG and showed cGMP stimulation by DEANO.

- Frankenreiter S, et al (Cardiovasc Res. 114(6):822-829, 2018) evaluated ischemia/reperfusion injury in mice with a cardiomyocyte-specific deletion of NO-GC (CM NO-GC KO) and in control siblings (CM NO-GC CTR) subjected to an in vivo model of AMI. Their studies demonstrated an important role of CM NO-GC for the cardioprotective signalling following AMI in vivo. The

authors concluded that CM NO-GC function is essential for the beneficial effects on infarct size elicited by pharmacological elevation of cGMP

Ad 1) The references suggested by the reviewer that speak in favor of the existence of NO/cGMP signaling in cardiomyocytes are now mentioned and cited in the revised manuscript (page 12 lines 2-3).

In addition, we included the results slices from mice in which the major GC isoform GC1 had been deleted specifically in cardiac fibroblasts and yielded results compatible with the absence of the cGMP transfer indicating that cardiomyocyte cGMP originates from fibroblasts. The results are described on page 11 lines 7-19 and depicted in Fig. 8 D-F.

2. Menges et al must discuss that the drugs which they used to block Cx-43 are not specific. Hence, besides blockade of Cx 43, additional effects may have contributed to the inhibition of the cGMP response in cardiomyocytes.

In particular:

Carbenoxolone is a synthetic derivative of glycyrrhizic acid (from licorice root) which has many different actions: mineralocorticoid activity, stimulation of prostaglandin production, inhibitory effect on cell differentiation. Moreover, it inhibits the conversion of inactive cortisone to cortisol by blocking 11β-hydroxysteroid dehydrogenase (11β-HSD). Carbenoxolone is a modestly potent, reasonably effective, water-soluble blocker of gap junctions

Gap 26 is a peptide, corresponding to residues 63 - 75 of connexin 43, which is a gap junction blocker. But it also inhibits IP3-induced ATP release, without inhibiting gap junctional coupling in endothelial cells.

etc.

Ad 2) The non-specificity of the gap junction inhibitors is now discussed in the revised ms. Moreover, additional experiments with gap junction peptides were performed to confirm gap junction dependency so far only performed with CBX. We now present a Western Blot of WT cardiac slices showing that the peptide gap junctions inhibitors prevent the enhanced phosphorylation of PLN induced by 3 nM Iso and BAY41-2272 in cardiac slices as did CBX (new Fig. 8C lanes 6+7 vs lane 3). In addition, the peptide inhibitors did abolish PLN phosphorylation induced by 10 nM Iso as did CBX (lanes 8-10).

Moreover, also the enhancement of Iso-induced cAMP by BAY41-2272 measured in RIAs was prevented by the peptide gap junction inhibitors in cardiac slices (new Fig. 6 D+E).

The results are described on page 10 line 19-21 (pPLN Western blot) and page 8 line 21-25 (cAMP RIA).

We agree with the reviewer that both types of gap junction inhibitors have non-specific side effects; yet, the speculation that the inhibitory action on NO-induced cGMP in cardiac myocytes of all inhibitors should not be related to inhibition of gap junctions appears rather implausible.

3a. To overcome these pharmacological limitations, the authors used siRNA to downregulate Cx-43 in cultured cardiac fibroblasts and then co-cultured such cells with freshly isolated adult murine cardiomyocytes during 1-2 days. This experiment raises at least two questions: how efficient wass the generation of fibroblast-cardiomyocyte gap junctions in this short time?

Ad 3a) From the results of 12 % of NO-responsive cardiac myocytes compared to CNP-sensitive cells set as 100 %, we conclude that about 12 % of viable cardiomyocytes formed gap junctions to fibroblasts within the 1-2 days of the co-culture. With 1-5 h, the $t_{1/2}$ of gap junctions is rather short.

3b. How many adult murine cardiomyocytes remained viable after 1-2 days? This is not described in the manuscript. But the facts that the authors report the cGMP responses in "percent of CNP-responsive cells" and that overall from such CNP-responsive cells only 12% were responsive to NO, suggests that most of the cardiomyocytes were not viable. Could the content of the broken cardiomyocytes affect the NO/cGMP responses?

Ad 3b) Taking into consideration that gap junctions between fibroblasts and myocytes had to be newly formed during the culturing time of 1 or 2 days, we do not judge 12 % of CNP-responsive cells as very low. Furthermore, apparently a much higher number of myocytes were viable as judged by CNP stimulation.

We cannot envision how the content of the broken cardiomyocytes affects the NO/cGMP responses? However, the cGMP transfer can be observed in acute slices and is abolished by knock out of NO-GC specifically in fibroblasts.

4. How relevant is this fibroblast-cardiomyocyte crosstalk "in vivo"? As illustrates in Figure 1 of the manuscript, a healthy adult heart contains few fibroblasts (this figure illustrates about 10 fibroblasts scattered between 50 cardiomyocytes). Not only the "number" but also the

“contact area” might be a problem. It seems that even for the few “big” cardiomyocytes which are in direct contact with a “small” fibroblast, the area of direct cell-cell contact is very small. If NO-derived cGMP only difuses through this small space, it may not reach functional levels or targets within the big cardiomyocyte (in vivo).

Ad 4) We agree with the reviewer that fibroblasts are comparatively small compared to myocytes. Therefore, uniformly high cGMP concentrations occurring throughout the myocytes are not very likely but apparently PDE3 mediated cAMP degradation possibly occurs in compartments of cardiac myocytes adjacent to fibroblasts. The enhanced phospholamban phosphorylation caused by NO-induced cGMP is indicative of a functional relevance. We now present additional results with slices from Tcf21-GC1-KOs in which NO-GC1 has been deleted specifically in Tcf21 fibroblasts underlining the important impact of NO-GC1 derived cGMP from cardiac fibroblasts (see below).

5. I wonder whether the authors could validate their ex vivo data with an in vivo approach, for instance using mice with a fibroblast-restricted deletion of NO-GC (floxed NO-GC mice were previously generated in their group and are possibly available for such studies)

Ad 5) According to the suggestion of the reviewer, we generated Tcf21-NO-GC1-KO mice, in which the alpha1 subunit of NO-GC has been deleted specifically in Tcf21-expressing fibroblasts. In acute cardiac slices of these mice, phospholamban phosphorylation induced by 10 nM Iso and the synergistic action of 3 nM Iso and the NO-GC activator were greatly reduced demonstrating that NO-GC1 in cardiac fibroblasts is required for the observed cGMP effects on PLN phosphorylation in cardiac myocytes.

The results are described on page 11 lines 7-19 and depicted in Fig. 8 D-F.

Reviewer III

1. The conclusion that cGMP from fibroblasts increases cAMP levels through PDE3 inhibition is a bit bold based on the evidence the authors provide in the manuscript. This conclusion is clearly stated in the title and throughout the text. I agree that this is the most likely explanation but the results shown are only indirect evidence. Even though PDE3 regulates the cGMP levels in cardiomyocytes after NO-GC activation in fibroblasts, and both NO-GC activation and PDE3 inhibition increase cAMP levels after FSK/Iso stimulation independently, these are only indirect evidences that the effect of NO-GC on cAMP is through PDE3. Thus, the authors should do the same experiments comparing cAMP levels and PLB-phosphorylation after GSNO/B41 stimulation in presence of Cil+FSK/Iso (vs Cil+FSK/Iso without GSNO/B41 and vs FSK/Iso with GSNO/B41). If GSNO does not give a further increase in cAMP than FSK/Iso+Cil, this will provide better evidence that PDE3 is involved. If GSNO/B41 gives an increase above PDE3 inhibition, this indicates that other mechanisms could be involved.

Ad 1) We agree with the reviewer and performed the suggested experiments (PLN Western Blots and RIAs). In the presence of Cil and Iso, BAY41-2272-treatment did neither cause enhanced phosphorylation of phospholamban nor further cAMP increases. The cAMP results are shown in the new Fig. 6F and described on page 8 lines 25-30. The PLN results are depicted in Fig 8C, lanes 2-5 and described on page 10 lines 21-26 in the revised version of the manuscript.

2. The authors conclude that there are no other PDEs involved than PDE1 and PDE3 as IBMX give the same cGMP levels as the combined inhibition of PDE1 and PDE3 inhibition. PDE9 has also been shown to be expressed in cardiomyocytes and should at least been discussed. IBMX do not inhibit PDE9, thus the authors cannot rule out that PDE9 could also regulate the NO-GC-induced cGMP.

Ad 2) We agree with the reviewer and performed the sensitive FRET measurements with two PDE9 inhibitors to rule out that PDE9 does degrade NO-induced cGMP in cardiac myocytes. Both PDE9 inhibitors did not increase NO-induced cGMP in myocytes of cardia slices in FRET measurements. The results are shown in the new Fig. 4C and described on page 7 lines 13-17.

3. PDE1 is also a dual PDE, thus even though PDE1 has not been shown to be involved in cross-talk between cGMP and cAMP (to my knowledge) it should be considered.

Ad 3) We agree with the reviewer and performed the suggested experiment with the new PDE1 inhibitor. As ITI did not increase Iso-induced phospholamban phosphorylation, we

conclude that PDE1 does not degrade the pool of cAMP responsible for the respective phosphorylation. The results are shown in Fig. 8C lanes 11-12 (compare to lanes 2+3) and described on page 10 lines 26-29 in the revised manuscript.

4. 12% is a low number of cardiomyocytes that responds to GSNO (figure 3)- why is there so few cells that responds, are there not gap junctions between all fibroblasts and cardiomyocytes? This should at least be commented on. Will it be possible to visually show that there are not gap junctions between all cells or is it that GSNO do not increase cGMP in all fibroblasts?

Ad 4) In the co-culture model, it should be considered that fibroblast and myocytes are derived from different animals. Fibroblasts from WT are already grown for a week, subsequently, myocytes derived from FRET sensor mice are plated on top of the fibroblasts. After 1 or 2 days, NO-induced cGMP is measured in myocytes. No gap junctions between fibroblast and myocytes exist at the time of plating; the proteins like connexin 43 responsible for the formation of hemichannels have to be expressed and to be assembled. Then, the two hemichannels provided by either one of two neighbouring fibroblasts and myocytes have to connected in order to form the respective gap junction. In sum, we consider 12% of NO-sensitive cardiomyocytes in which the gap junctions had to be formed to connect with fibroblasts compared to 100 % viable (CNP-sensitive) myocytes not surprisingly low.

Minor:

5. Spell out abbreviations when first mentioned: cAMP, cGMP, CER…

Ad 5) We includes a list of abbreviations page 22, lines 11-25.

6. mGC – not a common name for the membrane bound GCs. A more common name is the particulate GC (pGC)

Ad 6) We agree about the term mGC and according to the IUPHAR changed mGC to transmembrane guanylyl cyclases without abbreviating it.

7. NO-GC – not the most common name for the soluble GC. Maybe use sGC instead?

Ad 7) Again, we consulted the IUPHAR, which only gives GC-1 and GC-2 as abbreviations for 'Nitric oxide (NO)-sensitive (soluble) guanylyl cyclases'. We avoid the term soluble as at least GC-2 has been shown to be targeted to certain subcellular domains via its interaction with PDZ domains and therefore consider NO-GC a good abbreviation.

8. More details on incubation/stimulation times should be provided either in the methods or figures.

The respective information is given in the methods section for FRET measurements, radioimmunossays and PLN Western blot analyses.

9. What is the definition of CER (figure 2 and 4)?

Ad 9) Change of emission ratio (% CER), this is now explicitly stated in the methods section (page 19 line 14).

10. Figure 3B. The fraction of how many cells that responded to NO is shown from 5 experiments. How many cells in each experiment and condition? This is important information if there are fewer cells measured in those with siRNA.

We checked the number of CNP-responsive cells in a viewing field and it was not different in the siRNA transfected conditions (approx. 20 under each condition).

11. Figure 4. I agree that IBMX is broad, but maybe specify which PDEs that are inhibited as not all PDEs are?

Ad) 11 Now, the information that PDE9 is not inhibited by IBMX is given in the ms. and as already mentioned (ad 2), the impact of PDE9 is tested with 2 inhibitors. The results are shown in the new Fig. 4C and described on page 7 lines 13-17.

12. The concentration of cilostamide used is higher than needed and recommended based on selectivity. 1 µM should be enough. PDE2 and 5 will also be affected at 10 µM according to Sudo et al, Biochemical Pharmacology, 2000. This would probably not change the results in this study as PDE2 and PDE5 inhibition did not do much, however it should be taken into consideration.

Ad 12) In the newly performed experiments suggested by the referee, cilostamide was used at a concentration of 1 µM.

REVIEWERS' COMMENTS:

Reviewer #1 (Remarks to the Author):

The authors have addressed all my comments satisfactorily. I am happy to recommend their ms for publication

Reviewer #2 (Remarks to the Author):

In this studies of freshly prepared murine cardiac slices, Menges et al. extended their previous studies in co-cultured cardiac myocytes and fibroblasts which indicated that NO-induced cGMP formed in cardiac fibroblasts enters cardiac myocytes via gap junctions, thereby turning cGMP into an intercellular signalling molecule (Ref. 11 of the present manuscript). Here, the authors prepared ventricular slices from mice with cardiomyocyte-specific expression of a cGMP-FRET sensor. They show that application of NO increases cGMP levels in cardiomyocytes. These responses were abolished by connexin (Cx) 43 inhibitors, suggesting a Cx-43-dependent transfer of cGMP from non-myocytes (probably fibroblasts) to myocytes. Furthermore, the studies by Menges et al confirm that in cardiomyocytes, "paracrine" cGMP inhibits the dual substrate esterase PDE3A and thereby augments the cAMP-responses induced by isoprenaline. Ultimately, this improves cAMP/PKA-dependent phospholamban phosphorylation.

To corroborate such experiments in cardiac slices, the authors performed experiments in co-cultured Cx-43-deficient cardiac fibroblasts and cardiomyocytes. These experiments also indicated that the NO-dependent increases of cGMP in cardiomyocytes require the presence of Cx-43, suggesting that NO stimulates soluble guanylyl cyclase-dependent cGMP production in non-myocytes and transfer of cGMP to neighboring cardiomyocytes via Cx43. As shown by novel experiments included in the revised manuscript, the responses are also abolished when using cells or slices from mice with fibroblast-restricted deletion of NO-GC. This is a very convincing experiment and I hope that the authors will use these mice for future in vivo experiments to get further insights into the (patho)physiological impact of this "intimate" fibroblast-to-myocyte" communication. I fully understand that these experiments could not be done for the present manuscript.

These elegant studies are nicely designed, the experiments are well conducted, and the manuscript is very clearly written and easy to follow. The results are presented in a comprehensive way. I am sure that these original observations of a very special, intimate, and beneficial communication between fibroblasts and cardiomyocytes will be very interesting to many researchers working in cardiovascular pathophysiology and cyclic GMP signalling. The authors made a "tour de force" with new experiments, which convincingly answer the questions of the three reviewers.

Reviewer #3 (Remarks to the Author):

The authors have addressed all my concerns and I do not have further comments.
